# Thermo-responsive Bioink for Personalized 3D Printed Scaffolds with Antioxidant and Fibroblast Delivery to Accelerate Diabetic Wound Healing

**DOI:** 10.34133/bmr.0216

**Published:** 2025-06-11

**Authors:** Jisun Kim, Jiyeon Lee, Jung-Kyo Cho, Ki Wan Bong, Soo-Chang Song

**Affiliations:** ^1^ Center for Biomaterials, Biomedical Research Institute, Korea Institute of Science and Technology (KIST), Seoul 02792, Republic of Korea.; ^2^ Department of Chemical and Biological Engineering, Korea University, Seoul 02841, Republic of Korea.; ^3^ Division of Bio-Medical Science & Technology, KIST School, University of Science and Technology (UST), Seoul 02792, Republic of Korea.; ^4^ Nexgel Biotech, Co., Ltd., Hanam 12939, Republic of Korea.

## Abstract

Three-dimensional (3D) bioprinting is a promising field in tissue engineering, and the mechanical properties and biocompatibility of bioinks are essential factors. This study introduces a biocompatible, thermo-responsive poly(organophosphazene)-based bioink with excellent mechanical properties that provides effective drug release. First, we synthesized the Tyr-PPZ polymer, which contained an isoleucine ethyl ester, amino-methoxy poly(ethylene glycol), and tyramine. The Tyr-PPZ polymer was dissolved in phosphate-buffered saline to prepare TP bioink. The presence of hydrophobic components facilitated the homogeneous diffusion of caffeic acid into the bioink and conferred antioxidant properties. The PC bioink, prepared by incorporating caffeic acid into TP bioink, not only exhibited stable antioxidant properties but also showed excellent extrudability and printability due to its shear-thinning and recovery properties, which enabled the fabrication of various 3D scaffolds. Printed 3D scaffolds maintained high mechanical properties at body temperature (37 °C), which ensured scaffold stability for 30 d without additional cross-linking. In addition, to enhance diabetic wound healing through antioxidant properties and fibroblast delivery, PCC bioink was formulated by loading fibroblasts into PC bioink. Three-dimensional scaffolds fabricated using PCC bioink exhibited high cell viability for 7 d and promoted tissue regeneration in diabetic mice. In addition, PCC bioink provided antioxidant effects and accelerated wound closure, thick granulation tissue formation, and angiogenesis. This technology is promising as a next-generation bioink platform for diabetic wound treatment through a high-resolution 3D bioprinting scaffold that effectively delivers antioxidants and fibroblasts.

## Introduction

Bioinks are a key component of 3-dimensional (3D) bioprinting and provide a microenvironment that supports cell survival, proliferation, and differentiation by integrating biocompatible materials with living cells to form a 3D scaffold [1]. Bioinks used for bioprinting must have suitable shear-thinning properties. In extrusion bioprinters, the structural network of a bioink is temporarily broken down and recovers when subjected to high shear stress during extrusion to protect encapsulated cells from shear stress [2]. In addition, the bioink must have self-healing properties. The broken network must rapidly re-form when passing through the nozzle to provide a high printing resolution and a stable scaffold structure. The bioink must be biodegradable and biocompatible so that it does not cause any immune response in the body [3]. Finally, a bioink must also provide a biological microenvironment that supports cell survival and function. Therefore, an ideal bioink must have (a) appropriate shear-thinning properties, (b) sufficient mechanical strength for high resolution, and (c) excellent biocompatibility to ensure cell survival.

Hydrogel-based bioinks are widely used due to their high water contents, various biophysical properties, and abilities to mimic the extracellular matrix (ECM) [4]. Representative hydrogel bioinks include gelatin, hyaluronic acid, and collagen-based bioinks, which have demonstrated high biocompatibility but have low printability and mechanical strength, which limits the structural maintenance and durability of scaffolds [5]. To improve this, methacrylate-modified gelatin and collagen have been widely utilized as bioinks [6]. These bioinks exhibit excellent mechanical strength and minimal swelling under physiological conditions, which facilitates the fabrication of stable 3D structures. However, these bioinks have limited in vivo applicability, since their hydrogel network relies on ultraviolet cross-linking, which involves potentially toxic photoinitiators and poses challenges in tuning the degradation rate [7]. To overcome these limitations, thermo-responsive bioinks have been proposed as an alternative. For example, bioinks based on *N*-isopropylacrylamide (PNIPAM) and Pluronic F127 exhibit sol–gel transition characteristics depending on temperature changes, making them easy to apply in vivo without additional cross-linking processes [8]. However, their low mechanical strength and limited biological activity make them difficult to use for long-term 3D cell culture and in vivo applications [3,9]. To improve this, various attempts have been made recently to improve biocompatibility by utilizing combinations such as alginate-grafted-PNIPAM/methylcellulose [10] or PNIPAM with poly(acrylic acid) or gelatin [11] and chitosan-modified PNIPAM [12]. However, they still have limitations in terms of the long-term stability of the scaffold due to low mechanical strength. Therefore, a new type of thermo-responsive bioink with both high mechanical strength and biocompatibility is needed. To this end, we propose the use of poly(organophosphazene) (PPZ) polymers as bioink materials. A recent study reported that PPZ-based bioinks are effective for bone regeneration [3]. However, in that study, high mechanical strength was achieved by adding laponite, but cells were not directly loaded into the bioink. PPZ polymers are known to have not only thermo-responsive properties but also high mechanical strength and excellent biocompatibility [13,14]. Therefore, PPZ polymers are expected to be utilized as an ideal bioink that can directly load cells with high mechanical strength and excellent printing resolution.

Three-dimensional scaffolds fabricated through 3D bioprinting are used in various medical fields such as bone and cartilage regeneration and are also attracting attention in the skin regeneration field [15]. In particular, customized 3D scaffolds can be used to completely cover irregular and deep wound areas to prevent infection and effectively heal wounds by supplying moisture [16]. Diabetic wounds are difficult to treat due to a lack of nutrients and oxygen caused by hyperglycemia and reactive-oxygen-species (ROS)-induced oxidative stress [17]. Notably, oxidative stress impedes the differentiation of M1 into M2 macrophages, which results in a chronic inflammatory environment in diabetic wounds [18]. Therefore, advanced treatments that can modulate ROS levels, reduce inflammation, and promote angiogenesis are needed to encourage tissue regeneration in chronic wounds. Customized bioink scaffolds can deliver bioactive substances directly to wounds and support tissue regeneration [19]. Furthermore, antioxidants can crucially remove ROS, reduce oxidative stress, and promote cell survival [17]. Previous studies have shown that reducing ROS levels can promote the differentiation of M1 macrophages into M2 macrophages, alleviate chronic inflammation, and promote tissue regeneration [20]. Caffeic acid (CA) is a natural antioxidant found in edible plants and has attracted attention as a candidate diabetic wound healing promoter due to its ROS scavenging and anti-inflammatory effects [21]. The phenolic hydroxyl of CA acts as an electron donor and reduces free radical levels, relieves oxidative stress, and improves oxidative environments at wound sites [22]. CA also promotes wound healing by inhibiting the production of inflammatory cytokines [19]. Based on these reports, we aim to promote diabetic wound healing by CA in a thermo-responsive hydrogel bioink to continuously release antioxidants into the diabetic wound site. Fibroblasts also play an essential role in ECM formation and tissue regeneration and differentiate into myofibroblasts in damaged tissues to induce angiogenesis and support immune regulation [23]. Thus, we considered that a bioink containing CA as an antioxidant and fibroblasts might provide a suitable treatment for diabetic wounds by simultaneously reducing ROS levels, providing inflammation relief, and promoting angiogenesis in diabetic wound sites.

**Fig. 1.**
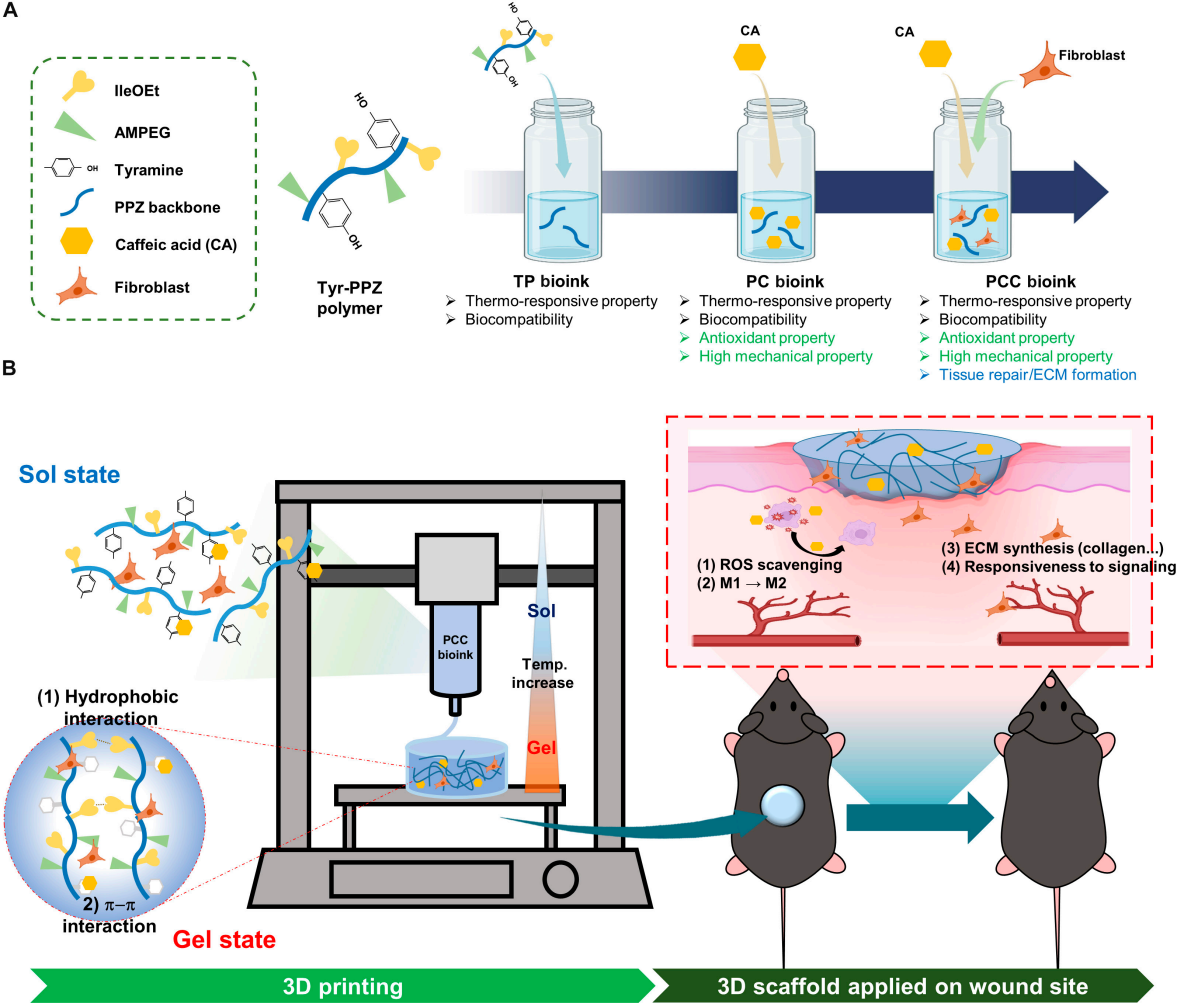
Schematic illustration of the preparation process of PCC bioink, the 3-dimensional (3D) bioprinting system using PCC bioink, and the diabetic wound healing process using the printed scaffold. (A) The process of preparing PCC bioink from the Tyr-PPZ polymer to TP bioink, PC bioink, and finally PCC bioink. All bioink components, including antioxidants and cells, are incorporated into the bioink through simple mixing below the gelation temperature (*T*_0_). (B) The temperature-responsive PCC bioink exists in a sol state in the nozzle and a gel state in the printing bed, enabling the immediate formation of 3D structures. The printed 3D scaffolds are applied on the wound site of diabetic mice; the chronic wound healing process is demonstrated due to the release of bioactive molecules from the bioink. Tyr, tyramine; PPZ, poly(organophosphazene); IleOEt, l-isoleucine ethyl ester; AMPEG, α-amino-ω-methoxy poly(ethylene glycol); ECM, extracellular matrix; ROS, reactive oxygen species.

In this paper, we describe the development of a 3D bioprinting scaffold optimized for diabetic wound healing based on a thermo-responsive PPZ bioink containing antioxidants and fibroblasts (PCC bioink) (Fig. 1). To improve the mechanical properties and biological properties of the thermo-responsive polymer, a novel polymer (Tyr-PPZ) was synthesized by adding tyramine (Tyr), and it was dissolved in phosphate-buffered saline (PBS) to prepare TP bioink. Subsequently, CA, an antioxidant, was added to the TP bioink to prepare PC bioink. By introducing Tyr into Tyr-PPZ and increasing its hydrophobicity, CA was uniformly mixed within the hydrogel, allowing for the development of a bioink with high mechanical properties through various interactions, without the need for additional additives or cross-linking processes. Finally, fibroblasts were loaded into the PC bioink to complete the PCC bioink. The mechanical properties of the thermo-responsive PCC bioink can be adjusted by controlling the temperature. In detail, below the gelation temperature (*T*_0_), antioxidants and cells can be easily mixed into the bioink, and at room temperature (bioink extrusion temperature), it maintains appropriate mechanical properties to enhance the viability of loaded cells and realize a high printing resolution. In addition, at body temperature (physiological environment), the high mechanical properties enable the structure to be stably maintained for a long time without an additional cross-linking process. As a result, PCC bioink has a high printing resolution and long-term structure retention ability, and its excellent biocompatibility ensures the long-term survival of the loaded fibroblasts and provides stability in vivo without toxicity, inflammation, and undesirable side effects. We evaluated the diabetic wound healing effect of PCC bioink through macrophage differentiation, wound healing process monitoring, and histological analysis, showing that a bioink containing antioxidants and fibroblasts can offer new possibilities for diabetic wound healing.

## Materials and Methods

### Materials

Hexachlorocyclotriphosphazene and l-isoleucine ethyl ester hydrochloride (IleOEt·HCl) were purchased from Sagechem (China) and Angene Chemical (UK), respectively. Methoxy poly(ethylene glycol) (750 Da), Tyr, CA, aluminum chloride (AlCl_3_), dimethylformamide, and 4-(dimethylamino)pyridine were purchased from Sigma-Aldrich (USA). Methoxy poly(ethylene glycol) was converted to α-amino-ω-methoxy poly(ethylene glycol) (AMPEG), as previously described [24]. Anhydrous tetrahydrofuran (THF), triethylamine (TEA), methanol, and *n*-hexane were purchased from Daejung (South Korea). Dulbecco’s PBS and Dulbecco’s modified Eagle’s medium (DMEM) were supplied by Welgene (Korea). Alpha-smooth muscle actin (α-SMA) and CD31 antibodies were purchased from Abcam (UK), CD206 antibody from Invitrogen (USA), and 4% paraformaldehyde from Biosesang (Korea).

### Synthesis of Tyr-PPZ

IleOEt, AMPEG, and Tyr were dried under vacuum at 40 °C for 3 d beforehand, and THF and TEA were prepared under anhydrous conditions in a N_2_ atmosphere. Briefly, hexachlorocyclotriphosphazene (10 g) and AlCl_3_ (0.5 g) were mixed in a glass ampule under Ar inside a glove box. The ampule was then sealed and heated at 250 °C for 5 h to synthesize poly(dichlorophosphazene). The synthesized poly(dichlorophosphazene) was weighed and then was sealed with a septum. The entire synthesis was performed under N_2_ using a cannula and Schlenk line to prevent moisture ingress. Dry IleOEt·HCl (24.82 g, 0.127 mol) was dissolved in 200 ml of anhydrous THF, and 30 min later, 100 ml of TEA was added. The reactor was then placed in a dry ice bath. The reaction was initiated by adding poly(dichlorophosphazene) (10 g) dissolved in THF to the reactor using a cannula. The reaction was allowed to proceed at room temperature for 3 h, and then the temperature was increased to 45 °C for 2 d. The reactor was cooled to room temperature, and AMPEG (14.88 g, 0.019 mol) dissolved in 50 ml of anhydrous THF was added, and the reaction was continued at 50 °C for 1 d. The next day, the reactor was cooled to room temperature, and Tyr (5.31 g, 0.038 mol) dissolved in 100 ml of dimethylformamide was added, and the reaction was completed by heating at 40 °C for 3 d. The mixture was then filtered through a glass filter and evaporated to remove side products and solvents. The product was purified by *n*-hexane precipitation for 1 d and subjected to methanol dialysis for 4 d and then deionized water dialysis for 4 d (molecular weight [MW] cutoff: 12 to 14 kDa). The purified polymer was filtered using a syringe filter (polypropylene, 0.45-μm pore size) and lyophilized to remove water. The obtained Tyr-PPZ was stored in a freezer.

### Characterization of Tyr-PPZ

The chemical structure of the polymer was analyzed using a Bruker Advance III HD 400-MHz nuclear magnetic resonance (NMR) spectrometer (Bruker, Germany) in CDCl_3_. The weight-average MW of Tyr-PPZ was measured using a gel permeation chromatography system (Agilent, 1260 Infinity II) equipped with a refractive index detector and a gel column (Agilent, PLgel 5 μm MIXED-C). THF containing 0.1 wt% tetrabutylammonium bromide was used as the mobile phase, and the analysis was conducted at 35 °C at a flow rate of 1 ml/min. Polystyrene standard samples with MWs of 6,570,000, 885,000, 479,200, 194,500, 75,050, 22,290, 10,330, 4,880, 1,210, 580, and 162 g/mol were used for the analysis.

### Preparation and characterization of the TP, PC, and PCC bioinks

TP bioink was prepared by dissolving Tyr-PPZ in PBS at a concentration of 10 wt% and storing it at 4 °C. To prepare PC bioink, CA (0.5 wt%) was added to the TP bioink and gently stirred at 4 °C for 20 min. PCC bioink was prepared by incorporating NIH3T3 cells into the PC bioink. To fabricate PCC bioink, NIH3T3 cells (1 × 10^6^ cells/ml) were centrifuged at 1,500 rpm for 5 min to obtain cell pellets. Then, PC bioink was carefully added onto the cell pellet and gently mixed by pipetting to ensure homogeneous cell dispersion. All bioink samples were prepared under ice bath conditions. The π–π interactions between the polymer and CA were analyzed using 2-dimensional nuclear Overhauser effect spectroscopy (2D NOESY) NMR (Bruker Avance NEO 600 MHz, Bruker, Germany) in CDCl_3_. Particle size analysis was performed using Dynamic light scattering (Malvern, UK). All bioink samples were diluted to 1 wt% in Dulbecco’s PBS, and measurements were conducted in triplicate at 25 °C.

### Cryo-FIB/SEM imaging

Cryo-focused ion beam scanning electron microscopy (cryo-FIB/SEM) was conducted using a Quanta 3D FEG unit (Thermo Fisher Scientific, USA) equipped with an Alto 2500 cryo-transfer system (Ganta, UK). TP, PC, and PCC bioinks were prepared in solution and gelation states. Samples were rapidly immersed in liquid nitrogen, and 1 s later, the freezing chamber was evacuated. Samples were then directly transferred to a preparation chamber at −190 °C pre-evacuated to 10^−5^ mbar. Metal deposition was conducted by plasma sputtering with a 10-mA current for 60 s. The metal-coated samples were then moved to a microscope chamber, also pre-evacuated to 10^−5^ mbar and precooled to −190 °C. Cryo-SEM images were acquired using a 5-keV electron beam and an electron current of 11.8 pA. FIB milling was performed with a 30-keV gallium ion beam and an ion current of 1 nA [25].

### Rheological analysis

Bioink samples were prepared at 4 °C, and rheological analysis was conducted using a rheometer (MCR102, Anton Paar) with a 25-mm parallel plate and a 0.5-mm gap. The thermo-responsive properties of the hydrogels were evaluated by measuring temperature-dependent storage and loss modulus from 5 to 60 °C at an oscillating frequency of 1 Hz and a strain of 10%. Viscosities were measured at increasing shear rates at 26 and 28 °C. Shear stress sweep was measured at 1-Hz stress and 26, 28, and 37 °C at increasing shear stress rates from 0.1 to 100 Pa. A self-healing test was conducted by applying alternating strains (3% and 30%) to hydrogels at a frequency of 1 Hz and temperatures of 26, 28, and 37 °C; measurements were taken 3 times.

### Three-dimensional printing method, printability, and structure stability testing

Three-dimensional printing was performed using a 3D bioprinter (BIO X, CELLINK, Sweden). The bioink was loaded into 3-ml cartridges purchased from CELLINK (Sweden). The temperatures of the nozzle (TP bioink: 28 °C/PC bioink: 26 °C/PCC bioink: 26 °C) and the printing bed (37 to 40 °C) were set according to each bioink. The temperature was controlled using the printer program; 3D printing was performed using a 3D bioprinter (BIO X, CELLINK, Sweden) at the optimal temperature and extrusion pressure. Various shapes were printed: stars (10 mm × 10 mm × 0.9 mm, 3 layers), cylinders (6 mm × 0.6 mm, 2 layers), grids (20 mm × 20 mm × 3 mm, 10 layers; 10 mm × 10 mm × 0.6 mm, 2 layers), and letters (KIST [15 mm × 40 mm × 2.4 mm, 8 layers]; KU [15 mm × 30 mm × 2.4 mm, 8 layers]). All structures were printed at the optimized speed of 5 mm/s and extrusion pressure of 35 kPa. The printability was calculated using the following equation for 3D structures printed in a grid pattern:

$$\text{Printability}=\left(\pi /4\right)\times \left(1/C\right)=L^{2}/16A,$$ (1)

*C* represents circularity, *L* is the perimeter, and *A* is the mean area of squares in grids. Values were measured using the ImageJ software, and printability and grid line thickness were estimated 3 times.

### DPPH assay

2,2-Diphenyl-1-picrylhydrazyl (DPPH) solution (0.2 mM; Biozoa, Korea) was stored at 4 °C; 200 μl of the sample to be measured was lyophilized and then dissolved in 200 μl of methanol. For the assay, 200 μl of the hydrogel sample dissolved in methanol and 800 μl of the 0.2 mM DPPH solution were added to a 1.5-ml Eppendorf tube and reacted for 15 min in the dark. After the reaction, 200 μl of each sample was transferred to a 96-well plate, and the absorbance was measured at 517 nm using Flash Multimode Reader (Varioskan, Thermo Scientific, USA). DPPH scavenging (%) was calculated using the following equation:

$$\text{DPPH scavenging effect}\;\left(\%\right)=\left(A_{b}-A_{s}\right)/A_{b}\times 100,$$ (2)

where *A*_b_ is the absorbance of the blank and *A*_s_ is the absorbance of the hydrogel sample.

### H2DCFDA staining

RAW 264.7 cells (1 × 10^5^ cells/well) were seeded into 24-well plates (SPL, Korea) and cultured in an incubator (5% CO_2_, 37 °C) for 1 d. Cells were then treated with DMEM containing 10% fetal bovine serum (FBS), 1% penicillin/streptomycin (P/S), and lipopolysaccharide (LPS; 1 μg/ml) for 12 h when the medium was replaced with fresh DMEM containing 10% FBS and 1% P/S. The TP and PC bioink scaffolds were printed into cylindrical shapes (diameter 6 mm, height 0.6 mm) using a 3D printer. The printed TP and PC bioink scaffolds were loaded into a 24-well insert (Corning, USA) and incubated with RAW 264.7 cells for 1 d. For fluorescence staining, the medium was removed, and 500 μl of 2′,7′-dichlorodihydrofluorescein diacetate (H2DCFDA) solution (D399, Invitrogen, USA) was added to each well. After a 30-min incubation, cells were washed 3 times with warm PBS, and fluorescence was analyzed using a confocal laser scanning microscope (Carl Zeiss, LMS7000).

### Real-time polymerase chain reaction

RAW 264.7 cells treated with bioink were prepared using the same method as the H2DCFDA protocol above. RNA was extracted using TRIzol (Invitrogen, USA), and RNA concentrations were measured using a NanoDrop ND-1000 unit (Thermo Fisher, USA). Reverse transcription of messenger RNA (mRNA) into complementary DNA was performed using SuperScript VILO MasterMix (Invitrogen, USA). The mRNA expressions of inducible nitric oxide synthase (iNOS) and TNF-α (M1 macrophage markers) and Arg-1 and IL-10 (M2 macrophage markers) were measured using a QuantStudio 1 real-time polymerase chain reaction (RT-PCR) instrument (Applied Biosystems, CA). All primers were purchased from Cosmogenetech (Korea). The mRNA expression levels were normalized versus that of β-actin. All experiments were performed in triplicate, and gene expressions were quantified using the 2^−ΔΔCt^ method. Primer sequences are listed in Table S1.

### Folin–Ciocalteu assay

The release profile of CA was measured using the Folin–Ciocalteu assay. To prepare the samples, the PC bioink was 3D printed onto a cylindrical scaffold (diameter 6 mm, height 0.6 mm) in a 6-well plate. The scaffolds were then immersed in 5 ml of PBS and incubated at 37 °C. At predetermined time points (0, 1, 4, 7, 10, 14, 20, and 30 d), PBS samples were collected and stored at −20 °C. For analysis, 100 μl of the collected PBS was transferred to 2-ml Eppendorf tubes, followed by the addition of 200 μl of Folin–Ciocalteu reagent (Sigma-Aldrich, USA). The mixture was gently shaken for 10 min in the dark, after which 800 μl of 2% sodium carbonate solution was added. The samples were then incubated in the dark at room temperature for 1 h. Absorbance was measured at 765 nm using Flash Multimode Reader (Varioskan, Thermo Scientific, USA). The amount of CA was quantified using a standard curve generated from CA solutions of varying concentrations (0.009 to 0.625 mg/ml) in PBS.

### Cell viability test

Bioink biocompatibilities were confirmed by 3-(4,5-dimethylthiazol-2-yl)-2,5-diphenyltetrazolium bromide (MTT) analysis. TP and PC bioinks were freeze-dried and then dissolved in DMEM (without FBS) at 80 mg/4 ml. NIH3T3 cells (2 × 10^4^ cells/well) were seeded into a 96-well plate (SPL, Korea) containing DMEM supplemented with 10% FBS and 1% P/S and incubated in an incubator (5% CO_2_, 37 °C) for 1 d. The culture medium was then replaced with polymer solution diluted to various concentrations (100 to 5,000 μg/ml) with FBS-free DMEM. After 24 h, MTT solution (Invitrogen) was added to the cells and incubated in the dark in 5% CO_2_ for 3 h at 37 °C. The MTT solution was then replaced with 150 μl of dimethyl sulfoxide (Daejung, Korea), and absorbance was measured at 570 nm using Flash Multimode Reader (Varioskan, Thermo Scientific, USA). NIH3T3 cell viability (%) was calculated using the following equation:

$$\text{Cell viability}\;\left(\%\right)=\left(A_{s}/A_{c}\right)\times 100,$$ (3)

where *A*_c_ is the absorbance of controls and *A*_s_ is the sample absorbance.

The cell viability loaded into the PCC bioink was evaluated using the live/dead assay. The prepared PCC bioink was loaded into the bioink nozzle and printed in a grid pattern (10 mm × 10 mm × 0.6 mm) into a 6-well plate. Printing was performed using a 22G needle at an extrusion pressure of 35 kPa, a printing speed of 3 mm/s, and a nozzle temperature of 26 °C. The printed scaffolds were cultured in an incubator (37 °C, 5% CO_2_) using DMEM. The scaffolds were stained for 1 h at 37 °C using a live/dead kit (Invitrogen, USA) on days 0, 3, 5, and 7. Staining was performed according to the manufacturer’s protocol. Fluorescence images of the stained cells in the scaffolds were obtained using a confocal laser scanning microscope (Carl Zeiss, LMS7000).

### In vivo diabetic wound healing test

All animal studies were conducted after obtaining the approval of the Institutional Animal Care and Use Committee (IACUC) of the Korea Institute of Science and Technology (KIST), and the experimental protocol was also approved beforehand by the IACUC (2023-086). C57BL/6J mice (4 weeks old, male) were obtained from DBL (Korea). The mice were injected with streptozotocin (STZ; Glentham Life Science, UK) to induce the diabetic model. The STZ solution was prepared by dissolving STZ in sodium citrate buffer (pH 4.5, Biosesang, Korea). Mice received intraperitoneal injections of STZ (50 mg/kg) for 7 consecutive days and were given 10% sucrose water to prevent sudden hypoglycemia. Blood glucose levels were monitored weekly using a CareSens N blood glucose meter (i-Sens, Korea). Mice with blood glucose levels higher than 350 mg/dl 3 weeks after STZ injection were considered diabetic. These mice (7 weeks old, male, *n* = 4 per group) were randomly assigned to 4 groups: a nontreated control group, a TP bioink group, a PC bioink group, or a PCC bioink group. Dorsal hair was shaved, and a 6-mm biopsy punch was used to create a wound. A polydimethylsiloxane mold (10 mm × 10 mm × 2 mm) with an 8-mm hole was then sutured around the wound, and a cylindrical scaffold (diameter 6 mm, height 0.6 mm) was fabricated using a 3D printer and placed on the wound. Printing was performed using a 22G needle at an extrusion pressure of 35 kPa and a printing speed of 3 mm/s. The wound was then covered with Tegaderm (3M, USA) and a bandage. The wound healing process was observed for 14 d.

### Histological analysis of regenerated skin tissue

Collected wound tissue samples were fixed in 4% paraformaldehyde for 2 d at 4 °C. Paraffin-embedded tissues were fixed to create paraffin blocks and cut into 5-μm-thick slices using a microtome. Next, tissue slides were deparaffinized and rehydrated by immersing them in xylene; 100%, 90%, 80%, and 70% ethanol; and distilled water in that order. The sliced tissues were then stained with hematoxylin and eosin or Masson’s trichrome (MT). All staining procedures were performed according to the manufacturers’ protocols.

### Skin tissue immunohistochemical analysis

Immunohistochemical analysis was performed using the VECTASTAIN Elite ABC-HRP kit (PK-7200, Vector Laboratories, USA). Collected tissue paraffin blocks were sectioned at 5 μm, and sections were deparaffinized, rehydrated, and heated for 15 min at 100 °C in antigen solution (S3022, DAKO, Denmark). Samples were washed twice with PBS (1×, pH 7.4), blocked with BLOXALL blocking solution (SP-6000, Vector Laboratories, USA) for 10 min to inhibit peroxidase activity, blocked with 2.5% normal horse serum for 20 min, incubated with CD206 primary antibody (1:100, PA5-101657, Invitrogen) for 30 min, and washed twice with PBS. Slides were then treated with a secondary antibody (biotinylated universal antibody) and ABC reagent from the kit. Color was developed using a peroxidase substrate solution. Hematoxylin staining (H-3404, Vector Laboratories, USA) was then performed, and the slides were mounted with mounting solution (H-5501, Vector Laboratories, USA). Finally, slides were imaged under an optical microscope (Zeiss Axio Vert.A1, Germany).

### Skin tissue immunofluorescence analysis

Mouse skin samples were collected 14 d after bioink treatment, fixed with 4% formaldehyde for 2 d at 4 °C, and embedded to form paraffin blocks, which were sectioned at 5 μm. Sections were deparaffinized, rehydrated, and subjected to antigen retrieval by heating for 15 min at 100 °C using retrieval solution (S3022, DAKO, Denmark). Samples were washed 3 times with PBS (1×, pH 7.4), blocked with 1% bovine serum albumin solution for 1 h at room temperature, treated with primary antibodies, and incubated overnight at 4 °C. The primary antibodies used were anti-CD31 (1:200, ab28364, Abcam) and anti-α-SMA (1:100, ab7817, Abcam). The samples were then washed 3 times with PBS and then treated with secondary antibodies for 1 h. The secondary antibodies used were Alexa Fluor 488-conjugated goat anti-mouse immunoglobulin G (1 μg/ml, A21121, Invitrogen) and Alexa Fluor 680-conjugated goat anti-rabbit immunoglobulin G (1:500, A21109, Invitrogen). After washing sections 3 times with PBS, they were mounted using Fluoroshield histological mounting medium containing 4′,6-diamidino-2-phenylindole (F6057, Sigma) and imaged under a confocal microscope (Carl Zeiss, LSM7000).

### Statistical analysis

Significant differences between groups were assessed by one-way analysis of variance followed by Tukey’s multiple comparison test. The analysis was conducted using the GraphPad Prism 9.0 software. Statistical significance is indicated as *P ≤ 0.05, **P ≤ 0.01, ***P ≤ 0.001, ****P ≤ 0.0001, and ns, meaning not significant, and results are presented as mean ± standard deviation.

## Results and Discussion

### Synthesis and characterization of the Tyr-PPZ polymer

**Fig. 2.**
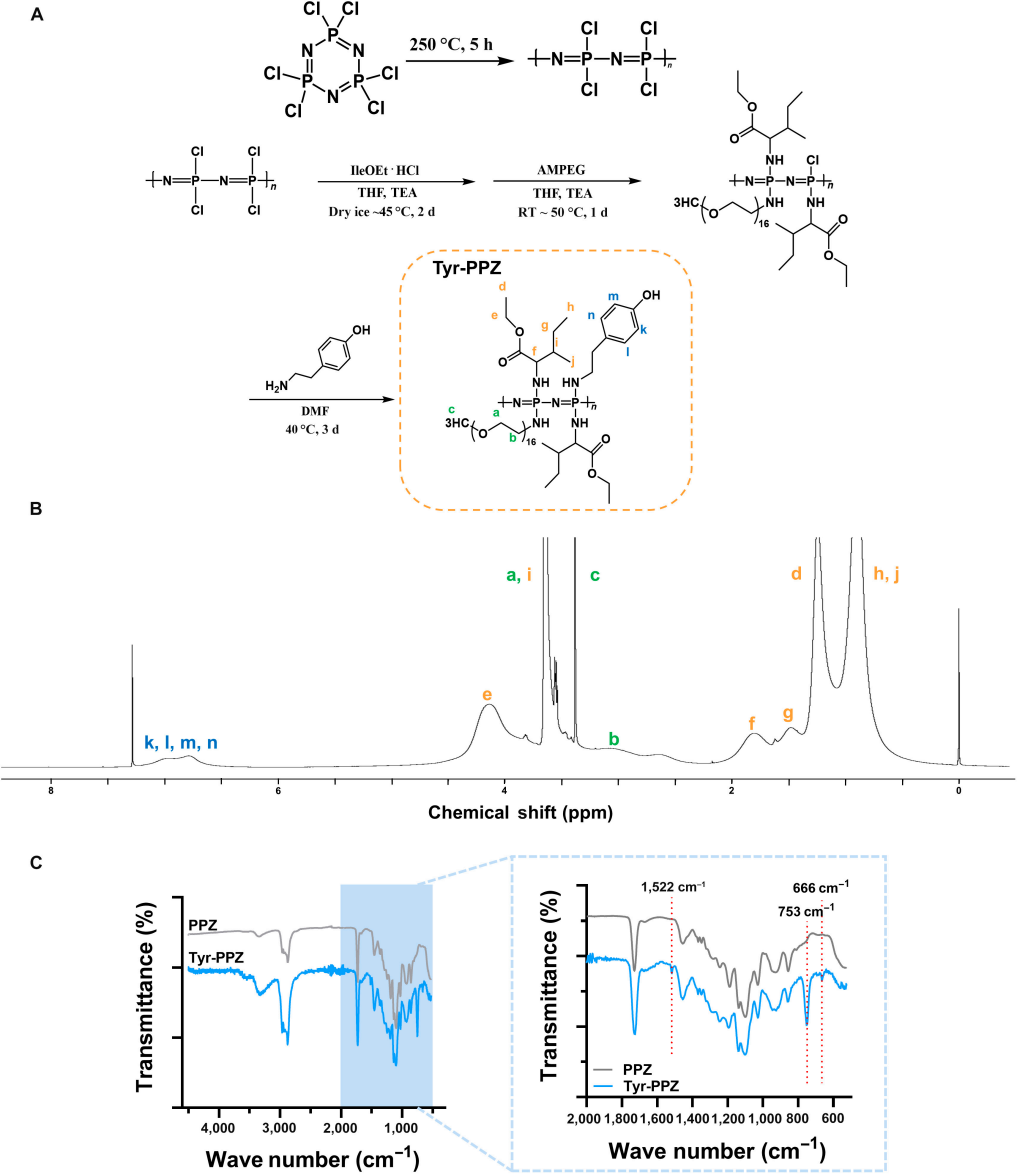
Synthesis and characterization of the Tyr-PPZ polymer. (A) Synthesis of Tyr-PPZ. (B) ^1^H nuclear magnetic resonance (NMR) spectrum of Tyr-PPZ. (C) Fourier transform infrared spectroscopy (FT-IR) spectra of PPZ and Tyr-PPZ. IleOEt·HCl, l-isoleucine ethyl ester hydrochloride; THF, tetrahydrofuran; TEA, triethylamine; RT, room temperature; DMF, dimethylformamide.

In this study, we synthesized Tyr-PPZ, a thermo-responsive polymer with excellent mechanical properties for use as a bioink, and the synthesis process is presented in Fig. 2A. In detail, PPZ containing Tyr (Tyr-PPZ) was prepared using a 3-step reaction, in which hydrophobic isoleucine ethyl ester (IleOEt), hydrophilic AMPEG, and Tyr were substituted into the PPZ backbone. Tyr is a naturally occurring amine commonly found in foods and beverages that possesses an aromatic ring and has a hydrophobic character [26]. We introduced the Tyr into the polymer chain for 2 reasons: (a) to facilitate the affinity between biologically active molecules containing an aromatic ring through π–π interactions and (b) to enhance the mechanical properties of Tyr-PPZ by introducing Tyr with hydrophobic properties. The Tyr-PPZ synthesized as described was used as the base material for the TP, PC, and PCC bioinks described below. The successful synthesis of Tyr-PPZ was confirmed by ^1^H NMR, which showed broad Tyr proton peaks appearing at 6.77 and 7.03 ppm. Substituent ratios of the synthesized Tyr-PPZ as determined by ^1^H NMR were as follows: IleOEt 79.5%, AMPEG 14.5%, and Tyr 6% (Fig. 2B). These ratios were optimized for the fabrication of bioinks with excellent mechanical properties by utilizing the characteristics of Tyr-PPZ, a PPZ-based polymer that induces sol–gel transition based on hydrophobic interactions. In addition, Fourier transform infrared spectroscopy (FT-IR) analysis was performed to confirm the successful synthesis of Tyr-PPZ. As a control, PPZ without a Tyr residue was used. The synthesis process of PPZ was performed in the same method as that in Fig. 2A, except for the Tyr conjugation step. Details of the synthesis process are described in the Supplementary Materials. FT-IR of Tyr-PPZ contained Tyr-specific peaks at 1,522 cm^−1^ (C=C aromatic ring), 753 cm^−1^ (C–H vibration), and 666 cm^−1^ (monosubstituted aromatic ring) [27–29], which were absent in the FT-IR spectrum of PPZ (Fig. 2C). The weight-average MW and polydispersity index of Tyr-PPZ, as determined by gel permeation chromatography, were 28 kDa and 2.5, respectively (Fig. S1).

### Preparation and characterization of the TP and PC bioinks

**Fig. 3.**
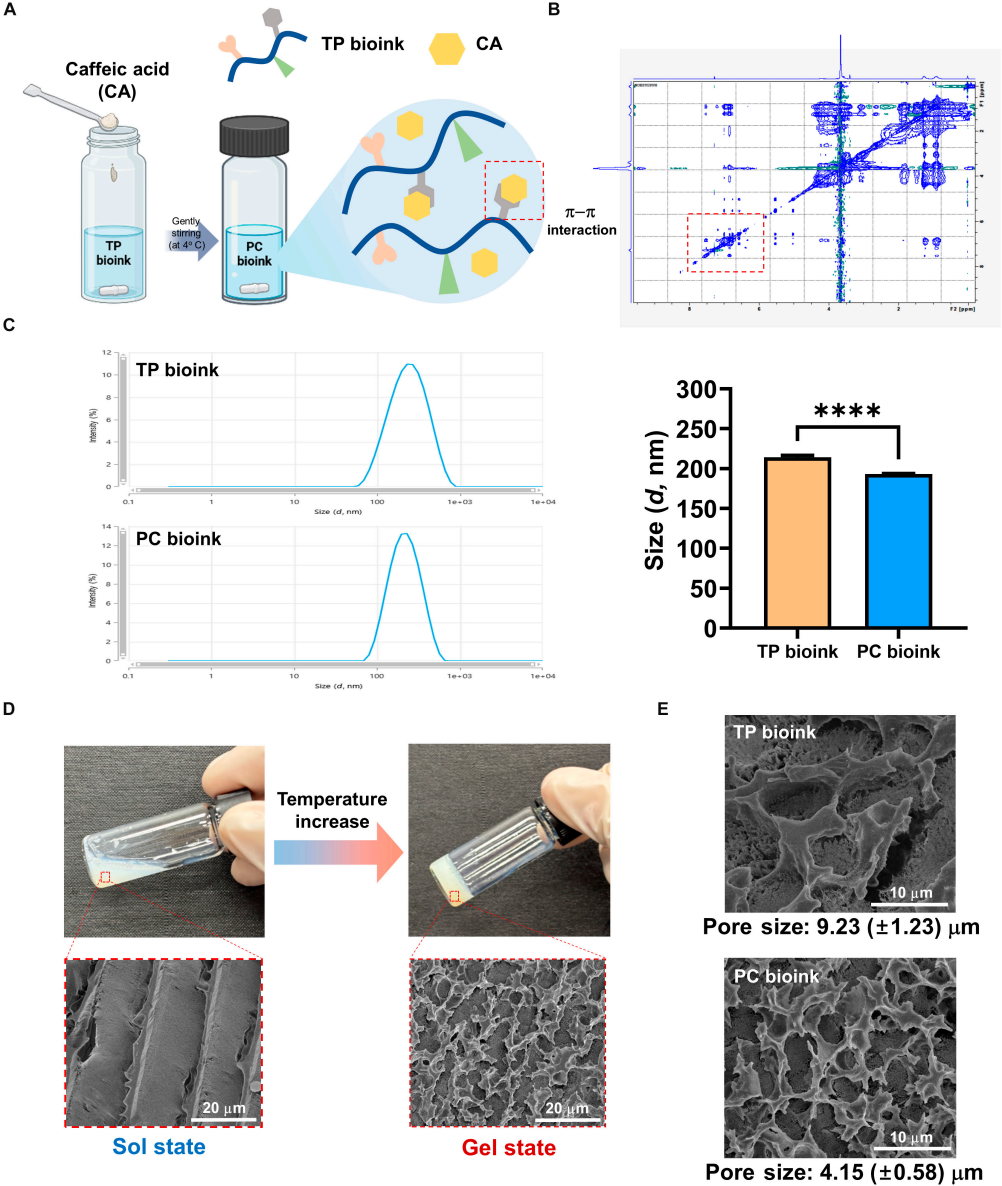
Preparation and characterization of the TP and PC bioinks. (A) Schematic illustration of the preparation of the PC bioink. (B) Two-dimensional nuclear Overhauser effect spectroscopy (2D NOESY) NMR spectra of the PC bioink. (C) Particle sizes and size distribution graph of the 2 bioinks (*n* = 3 each group). (D) Representative images of the sol–gel transition of the PC bioink and cryo-scanning electron microscopy (cryo-SEM) images of the PC bioink in the sol and gel states. (E) Cryo-SEM images of the TP and PC bioinks in the gel state.

The method for producing TP and PC bioinks using the synthesized Tyr-PPZ polymer is schematically shown in Fig. 3A. In detail, the TP bioink was prepared by dissolving the synthesized Tyr-PPZ in PBS at a concentration of 10 wt%, and the PC bioink was fabricated by simply mixing CA into the TP bioink solution. TP bioink was a translucent white solution, and after adding CA, PC bioink turned into a translucent pale yellow (Fig. S2). Homogenously dispersed CA powder was incorporated effectively into the TP hydrogel, presumably due to π–π interactions with the Tyr residues in the TP bioink or hydrophobic interactions. To verify this, 2D NOESY NMR analysis was performed, and cross peaks between TP bioink and CA were confirmed. The cross peaks showed the presence of shared protons between the 2 aromatic rings, proving that π–π interactions occurred effectively (Fig. 3B). The size and size distribution of hydrogel particles were evaluated by dynamic light scattering to confirm the stable loading of CA into the hydrogel. PPZ-based hydrogels can form micellar structures due to the presence of hydrophobic IleOEt and hydrophilic AMPEG [30]. The particle sizes of the TP and PC bioinks were 214.2 ± 2.1 and 193.3 ± 0.4 nm, respectively, and the smaller particle size of the PC bioink was attributed to the hydrophobic interaction between the bioink and CA (Fig. 3C). We previously reported that physical interactions between polymers and biological molecules can reduce particle size [14,31]. In addition, the observed single-peak particle size distribution suggests that CA was uniformly and stably loaded into the hydrogel network.

To determine the effect of introducing CA into TP bioink on the hydrogel network, the pore size change was analyzed by cryo-SEM. PC bioink was liquid at low temperature and did not form a hydrogel network (Fig. 3D). However, as the temperature increased, gelation occurred due to its thermo-responsive property, and a hydrogel network was formed through hydrophobic interactions between the hydrophobic groups present in the hydrogel. The porosity of the scaffold is considered an important property of bioink as it supports cell penetration and growth, facilitating waste removal and cell migration [32]. The pore sizes of the TP and PC bioinks were 9.23 ± 1.23 and 4.15 ± 0.58 μm, respectively (Fig. 3E). The pore structures of the TP and PC bioinks are similar, and the result that no aggregation was observed in the PC bioink suggests that CA was uniformly distributed within the TP bioink. In addition, the result that the pore size of the PC bioink was 45% smaller than that of the TP bioink indicates that CA enhanced the bonds between the polymer side chains through π–π interactions and hydrophobic interactions to form a denser gel network. Furthermore, the smaller pore size of the PC bioink could markedly affect the structural integrity of the hydrogel, suggesting its suitability as a bioink.

### Rheological analysis of the TP and PC bioinks

The thermo-responsive properties, mechanical properties, shear-thinning properties, and self-healing abilities of the TP and PC bioinks were measured at specific temperatures using a rheometer. These properties are essential for good printability, high resolution, and scaffold structure maintenance in extrusion-based bioinks [3,33]. As mentioned above, we aimed to fabricate thermo-responsive hydrogels with better mechanical properties by introducing Tyr into the PPZ backbone to increase gel hydrophobicity. Because low-critical-solution-temperature-based thermo-responsive hydrogels such as PPZ form gels due to interactions between hydrophobic groups, we believed that adding hydrophobic groups would improve the mechanical properties of bioinks [34]. Indeed, in our previous study, the maximum storage modulus of PPZ with an isoleucine and AMPEG ratio similar to the TP bioink was 2,209 Pa [3]. However, the maximum storage modulus of the TP bioink was 3,124 Pa, suggesting that the addition of Tyr resulted in a stiffer hydrogel. Nevertheless, unmodified TP bioink did not have sufficient mechanical strength, so we added CA to enhance its mechanical strength by providing additional interactions.

**Fig. 4.**
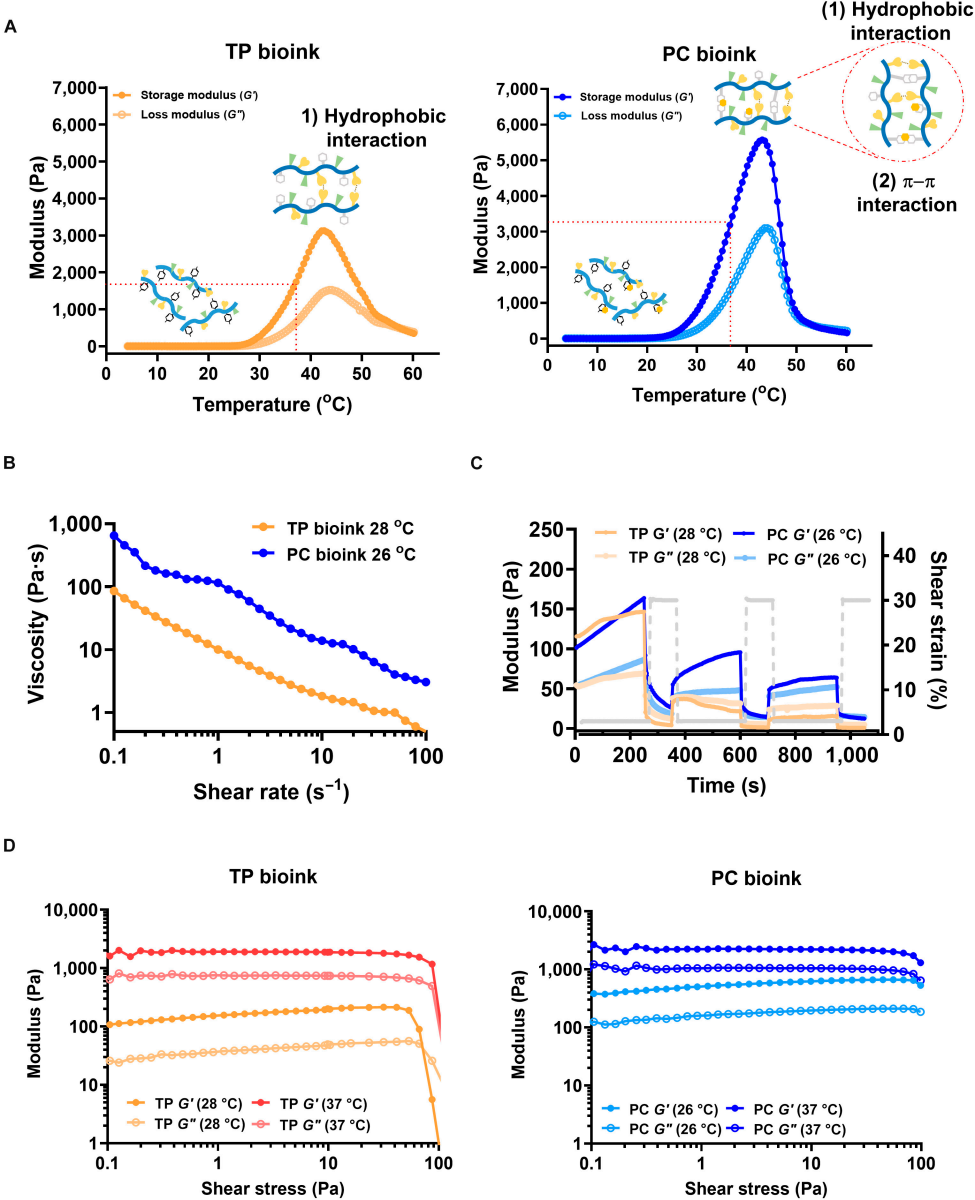
Rheological analysis of the TP and PC bioinks. (A) Temperature-dependent storage and loss modulus of TP bioink and PC bioink. (B) Shear-thinning properties of the TP and PC bioinks under shear rate sweeps. (C) Recovery properties of the TP and PC bioinks at 28 and 26 °C under high- (30%, 1 Hz) and low-strain (3%, 1 Hz) cycling (3 repetitions). (D) Storage and loss modulus of the 2 bioinks measured at optimized printing temperatures (TP bioink 28 °C and PC bioink 26 °C) and at body temperature (37 °C) under shear stress sweeps.

To confirm the thermo-responsive properties of the prepared bioinks, the temperature-dependent changes in the storage modulus of TP and PC bioinks were measured (Fig. 4A). For the TP bioink, the gelation starting temperature (*T*_0_) was 23.6 °C and the maximum storage moduli (*G*′_max_) were 3,124.2 Pa at 42 °C and 1,741.3 Pa at body temperature (*G*′_37°C_). In contrast, the gelation of the PC bioink started at 14.5 °C, and the maximum storage moduli were 5,573.2 Pa at 43 °C and 3427.3 Pa at 37 °C. These values indicate that the storage modulus of PC bioink at body temperature (*G*′_37°C_) increased by 55% and the maximum storage modulus (*G*′_max_) increased by 56% compared to those of TP bioink. This indicates that the synergistic effect of the hydrophobic interactions in the PC bioink and the π–π interactions formed by the added CA increased the storage modulus. The thermo-responsive properties of the TP and PC bioinks allow simple control of extrudability by adjusting the printing nozzle temperature, and their high mechanical properties at body temperature maintain the scaffold shape without an additional cross-linking process. In general, hydrogel-based bioinks cross-linked through physical interactions have weak mechanical properties and are not suitable for maintaining 3D printed structures without an additional chemical cross-linking process [35]. In this study, the PC bioink system overcomes this drawback without the use of cytotoxic chemical cross-linking processes. This bioink demonstrated excellent extrusion control and high-resolution retention, highlighting its potential for 3D printing applications.

To determine the extrudability and scaffold shape retention ability at body temperature, the shear-thinning behavior, self-healing ability, and shear stress sweep tests of TP and PC bioinks were perfomed. The extrusion 3D printing experiments using prepared bioinks showed that the optimal printing performance was achieved when the storage modulus was in the range of 100 to 150 Pa, and the evaluation temperature was set accordingly. The nozzle temperature determined based on the temperature-dependent rheological data of the storage modulus was 28 °C for TP bioink and 26 °C for PC bioink. Shear-thinning properties were evaluated using the optimal extrusion temperatures of the TP and PC bioinks (Fig. 4B). Both bioinks exhibited shear thinning, suggesting that they could be easily extruded at room temperature. In addition, to confirm the recovery property, high (30%) and low shear strains (3%) were applied alternately to the TP and PC bioinks (Fig. 4C). When bioinks are extruded, shear thinning occurs and temporarily destroys the hydrogel network, converting them into a sol state; thus, rapid network recovery is essential after extrusion to maintain a 3D scaffold shape [36]. After the application of a high shear strain, the TP bioink network was transformed into a sol state, whereas the mechanical properties of the PC bioink recovered and the bioink maintained a gel state even at a high shear strain, suggesting that the gel form of the PC bioink would be maintained during extrusion at printing. In addition, the self-healing ability of the PC bioink was demonstrated by subjecting it to multiple cycles of alternating shear strains. After a single application of a high shear strain, 58% of the original storage modulus was immediately recovered, and after the second application, 39% of the original value was recovered. The superior recovery of resistance ability after applying a high shear strain to the PC bioink resulted because adding CA increased network recovery through π–π interactions with Tyr in the polymer side chains [37]. Shear stress sweep tests were performed in the sol state (TP bioink 28 °C, PC bioink 26 °C) and gel state (both at 37 °C). The shear modulus of TP bioink decreased rapidly at high shear stress, whereas PC bioink remained stable even at high shear stress (Fig. 4D). This suggests that PC bioink has sufficient mechanical properties to withstand high shear stress in an environment similar to body temperature. In conclusion, PC bioink exhibits excellent shear-thinning and self-healing properties at extrusion temperature while maintaining high mechanical strength at body temperature to form stable gels. Therefore, thermo-responsive bioink is a very promising material for extrusion-based 3D bioprinting.

### Three-dimensional printability of the PC bioink

**Fig. 5.**
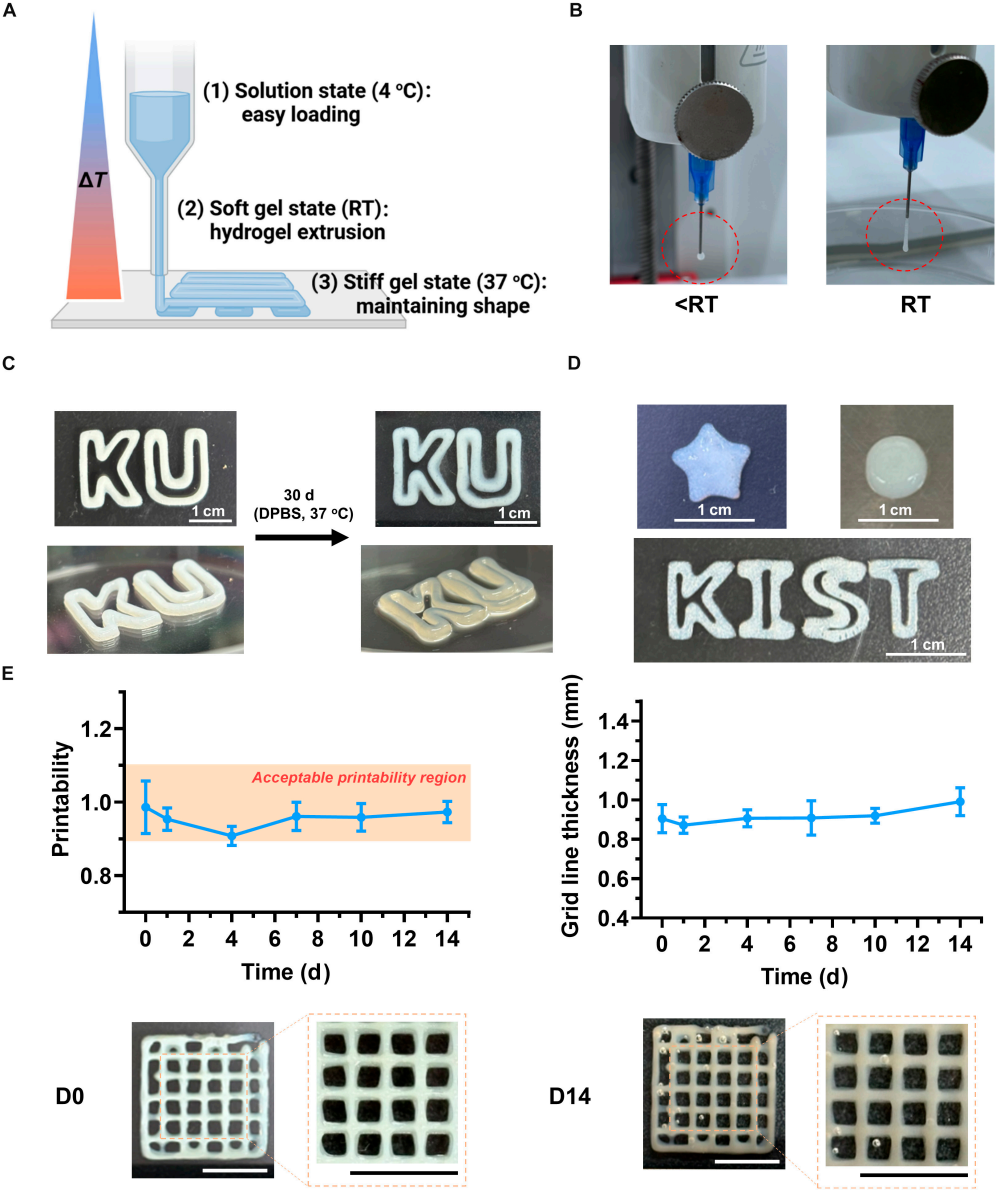
The PC bioink exhibited excellent printability when the printing nozzle and bed temperatures were appropriately controlled. All 3D structures were printed under optimized conditions: nozzle temperature of 26 °C, bed temperature of 40 °C, print speed of 5 mm/s, and extrusion pressure of 35 kPa. (A) Overall scheme of the 3D printing of the PC bioink. The illustration was created using BioRender.com. (B) Images of PC bioink extrusion at different nozzle temperatures. (C) A letter structure (“KU”) printed under optimized conditions, showing morphological changes observed after 30 d in phosphate-buffered saline (PBS) at 37 °C. (D) Various shapes (star, cylinder, and letters [“KIST”]) printed under optimized conditions. (E) Quantification of printability (refer to Eq. 1) and changes in grid line thickness over 14 d in PBS at 37 °C using the printed grid (*n* = 5 each group) and representative images of the printed grid scaffold at day 0 and day 14 (scale bar: 1 cm). Results are presented as mean ± standard deviation. DPBS, Dulbecco’s phosphate-buffered saline.

Three-dimensional printing using the thermo-responsive PC bioink exhibited temperature-dependent behavior. Specifically, at low temperatures (below *T*_0_, as determined using rheometer data in (Fig. 4A), the PC bioink existed in a liquid state and was easily loaded into the nozzle. In addition, the nozzle temperature was adjusted to confirm the extrusion temperature. Temperature-dependent rheometer data showed that the PC bioink existed as a soft hydrogel at printing temperatures of 24 to 26 °C, and at these temperatures, the bioink was smoothly extruded (Fig. S3). On reaching the printing bed at 37 to 40 °C, the storage modulus of the bioink increased due to its thermo-responsive properties, and the hydrogel was rapidly converted into a stiff gel with considerable mechanical strength, which maintained the 3D scaffold structure (Fig. 5A). To demonstrate this, images were taken to capture the extent of hydrogel extrusion at different nozzle temperatures. At temperatures below room temperature, the extruded PC bioink did not form a sufficient network, which resulted in droplet extrusion and failed printing. In contrast, at room temperature, a soft gel with appropriate mechanical properties was formed, and the bioink was smoothly extruded (Fig. 5B). Therefore, smooth printing was possible by controlling the mechanical properties of the PC bioink using temperature. The scaffolds printed in this manner maintained their shape even after being immersed in PBS at 37 °C for 30 d (Fig. 5C). This indirectly suggests that the printed scaffolds can maintain their shape for a long time even in a physiological environment. In addition, scaffolds with various shapes, such as star, cylinder, and letters, could be printed at a high resolution (Fig. 5D). The printability of PC bioink and its ability to maintain the printed shape were confirmed through the printed grid-shaped scaffold. Grid shapes and thicknesses were observed after immersing them in PBS at 37 °C for 14 d. Printability was calculated using the circularity, average area, and perimeter of the squares in the grid structure. It has been reported that acceptable printability is observed when calculated printability values are between 0.9 and 1.1 [38]. The calculated printability value of PC bioink was within the acceptable range of 0.90 to 0.98 for 14 d. Grid line thickness increased gradually from 0.90 ± 0.06 mm on the first day to 0.99 ± 0.06 mm on day 14, but no marked change was observed (Fig. 5E). This indicates that the aqueous environment does not markedly influence bioink resolution. To investigate the swelling and degradation behaviors of the TP and PC bioinks, the weights of 3D scaffolds were measured after immersing them in PBS at 37 °C for up to 14 d. The weights of the TP and PC scaffolds gradually increased by 121.9% ± 1.4% and 114.7% ± 2.5%, respectively, on day 14 (Fig. S4). This suggests that the hydrophobic interactions and π–π interactions due to CA in the PC bioink formed a denser network and reduced water absorption. These low swelling characteristics and high mechanical properties do not affect the resolution of the printed 3D scaffolds in a physiological environment and can provide sufficient time for sustained delivery of antioxidants and fibroblasts. In summary, the extrudability of the PC bioink was easily controlled by changing the nozzle temperature, and the bioink achieved a high printing resolution without the need for an additional process. In addition, the PC bioink has the potential to become practically useful because it resisted swelling in aqueous solution and maintained its original shape.

### ROS scavenging effect of antioxidants encapsulated in PC bioink

**Fig. 6.**
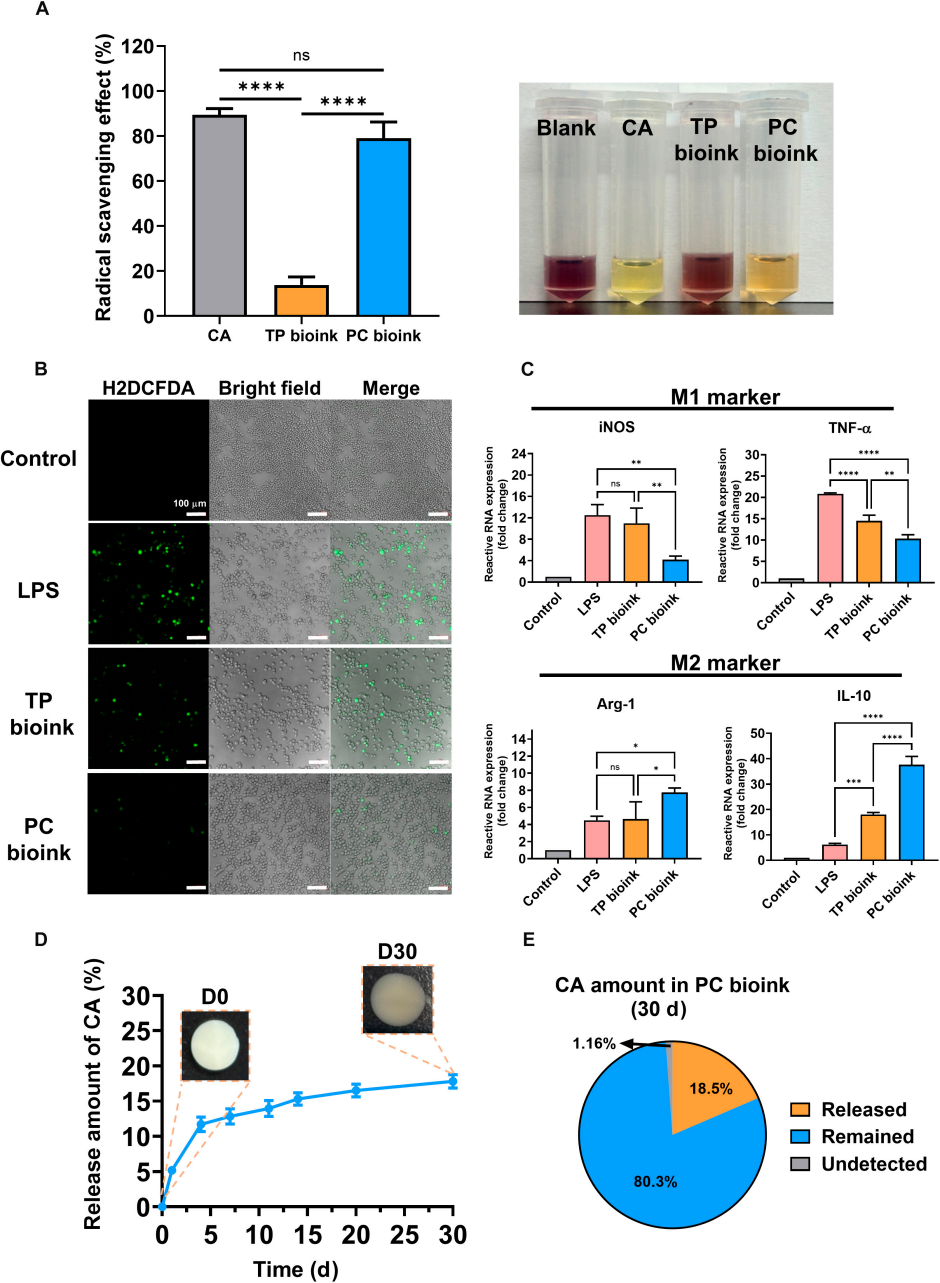
Antioxidant properties of the PC bioink. (A) Radical scavenging effect and colorimetric images of caffeic acid (CA) and TP and PC bioinks subjected to a 2,2-diphenyl-1-picrylhydrazyl (DPPH) assay (*n* = 3 each group). (B) Intracellular ROS scavenging effects of TP and PC bioinks as determined by an 2′,7′-dichlorodihydrofluorescein diacetate (H2DCFDA) assay (scale bar: 100 μm). (C) Relative gene expressions level of M1 and M2 polarization markers in RAW 264.7 cells treated with each bioink (*n* = 3 each group). (D) CA release profile from PC bioink after 30 d (*n* = 3 each group). (E) Amount of CA in a PC bioink scaffold after 30 d in PBS (*n* = 3 each group). Results are expressed as mean ± standard deviation. LPS, lipopolysaccharide; iNOS, inducible nitric oxide synthase.

Treatment strategies based on reducing ROS levels provide an effective method of healing diabetic wounds. Previous studies have shown that scavenging radicals, including ROS, induce the M2 polarization of macrophages and promote tissue regeneration [39]. The antioxidant effect of CA in PC bioink was demonstrated by DPPH assays (Fig. 6A). PC bioink showed a high radical scavenging ability of 79.0% ± 5.9%, which was similar to the radical scavenging ability of CA (89.4% ± 2.2%). On the other hand, TP bioink had a radical scavenging ability of only 13.8% ± 2.9%. Thus, CA was confirmed to exhibit effective radical scavenging ability even after being loaded into the bioink.

Furthermore, to confirm the effect of ROS scavenging at the cellular level, H2DCFDA staining was used to confirm the macrophage polarization effect mediated by PC bioink (Fig. 6B). The TP and PC bioink scaffolds used in the experiment were printed in a cylindrical shape (diameter 6 mm, height 0.6 mm) and applied to cells using a transwell. LPS-induced M1 macrophages exhibited green fluorescence, and fluorescence expression was observed using confocal microscopy [40]. The TP bioink scaffold without antioxidant properties exhibited green fluorescence at a level similar to that of LPS-treated cells, whereas low green fluorescence expression was observed in cells treated with the PC bioink scaffold. This suggests that the PC bioink scaffold may contribute to the differentiation of M1 macrophages into the M2 phenotype. To more clearly confirm the effect of PC bioink on inducing macrophage differentiation, RT-PCR was performed (Fig. 6C). The degree of macrophage differentiation was quantified using iNOS and TNF-α as M1 macrophage markers, Arg-1 and IL-10 as M2 macrophage markers, and β-actin as a housekeeping gene. No marked difference was observed between LPS-treated macrophages and TP-bioink-treated macrophages. However, the RNA levels of iNOS and TNF-α treated with PC bioink, which contains antioxidants, were 4.19 ± 0.54-fold and 10.32 ± 76-fold, respectively, which were significantly lower than those of the LPS-treated group. In addition, the RNA levels of Arg-1 and IL-10, which are M2 macrophage markers, were 7.76 ± 0.42-fold and 18.03 ± 0.65-fold, respectively. The RNA levels were significantly higher than those of the LPS-treated group. These results suggest that CA loaded in PC bioink can effectively scavenge radicals and induce differentiation into anti-inflammatory M2 macrophages. In conclusion, this indicates that PC bioink can be effectively applied to radical-rich environments such as diabetic wounds. To indicate the release profile of CA loaded into PC bioink, the printed scaffolds were immersed in PBS at 37 °C for 30 d (Fig. 6D and E). According to the release profile, 18.5% of CA was gradually released over 30 d, and 80.3% of CA was retained in PC bioink without an initial burst release. CA is known to exhibit antioxidant properties at low concentrations but is cytotoxic at high concentrations [41]. To reduce the risk of side effects on surrounding cells, the optimal concentration of CA in the PC bioink was set to 0.5 wt% based on its release profile. The sustained release profile of these PC bioinks suggests that they can exert effective antioxidant effects in a sustained manner while minimizing the impact on surrounding cells in in vivo implantation or treatment. These results demonstrate that CA was stably loaded into PC bioink, suggesting that PC bioink has long-term antioxidant properties.

### Biocompatibilities of the TP, PC, and PCC bioinks

**Fig. 7.**
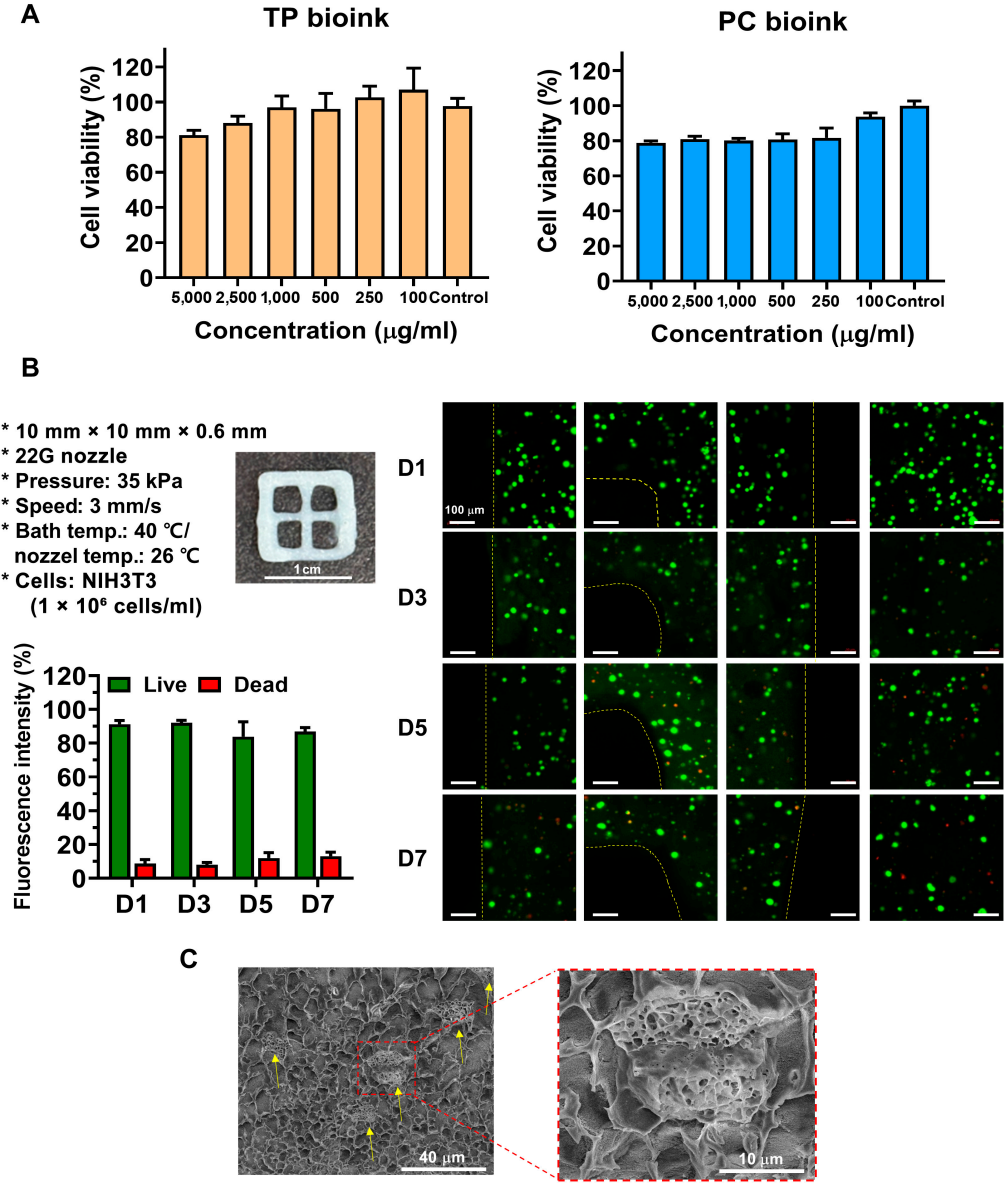
Biocompatibility of TP and PC bioinks and characterization of PCC bioink. (A) 3-(4,5-Dimethylthiazol-2-yl)-2,5-diphenyltetrazolium bromide (MTT) assay results for cell viability after treatment with various concentrations of TP and PC bioinks (*n* = 5 each group). (B) Three-dimensional scaffold printing images of PCC bioink, printing conditions, live/dead analysis images for 1, 3, 5, and 7 d using the printed scaffolds and fluorescence intensity analysis results at each time point. Fluorescence intensity was calculated using the ImageJ software (*n* = 4 each group; scale bar: 100 μm). (C) Cryo-SEM image of PCC bioink after thermal cross-linking (yellow arrows indicate fibroblasts).

Biocompatibility is a crucial factor of biomaterials. MTT assays and live/dead assays were conducted to evaluate the biocompatibility of the TP and PC bioinks. According to ISO guideline 10993-5, in MTT assay, a cell viability of over 80% is considered nontoxic, and one below 40% is considered toxic. MTT assay results showed that both bioinks exhibited minimal toxicity across various concentrations (Fig. 7A). This suggests that both TP bioink and PC bioink are suitable for bioapplication. Furthermore, to evaluate the cell viability within the bioink, PCC bioink loaded with fibroblasts in PC bioink was prepared. The prepared PCC bioink was used to fabricate a grid-shaped 3D scaffold using a 3D printer, and the scaffold was cultured for 7 d to confirm the viability of cells incorporated within the bioink (Fig. 7B). Cell viability was confirmed using a live/dead assay, and various regions of the printed scaffold were imaged using a confocal microscope. The scaffolds made with PCC bioink were extruded at a pressure of 35 kPa, which does not cause excessive stress to the cells, and maintained a stable shape for 7 d. As a result, approximately 85% of the cells were viable inside the 3D scaffold after 7 d, as confirmed by the confocal microscope images. In addition, the cryo-SEM images confirmed that the fibroblast cells were uniformly distributed inside the PCC bioink and stably encapsulated into the hydrogel network (Fig. 7C). These results demonstrate the high biocompatibility of the PCC bioink and suggest that the bioink loaded with fibroblasts can maintain its 3D shape without an additional cross-linking process, using its thermo-responsive property, and that the PCC bioink can be useful for in vivo cell delivery.

### In vivo wound closure in diabetic wounds treated with bioink

**Fig. 8.**
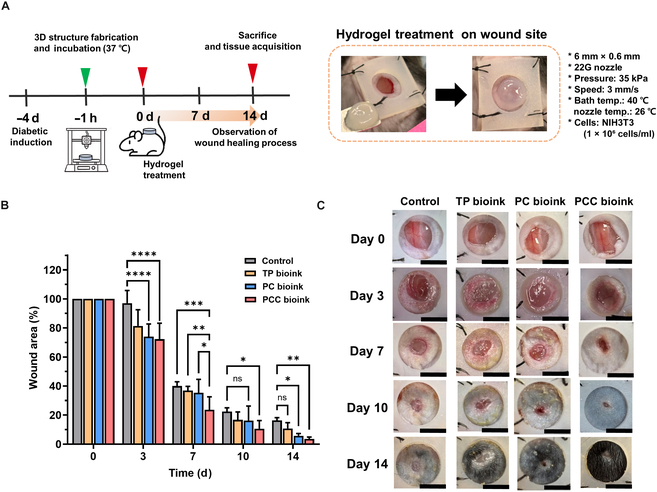
In vivo evaluation of the efficacy of bioink therapy in a mouse diabetic wound model. (A) Schematic illustration of the wound healing timeline. (B) Quantification of wound sizes after treatment for different times (*n* = 4 each group). (C) Representative photographs of wound sites over the 14-d treatment period (scale bar: 5 mm). Quantitation was performed using the ImageJ software, and results are expressed as mean ± standard deviation.

Based on the properties of PCC bioink, such as antioxidant properties, the oxygen radical scavenging effect, macrophage differentiation induction, and the fibroblast delivery mentioned in the previous in vitro experiments, the bioink proposed in this study was expected to effectively accelerate chronic wound healing. The effects of 3D scaffolds formed with each bioink on chronic wound healing were investigated using a diabetic mouse model. Chronic wound healing experiments were performed according to the schedule presented in Fig. 8A, and the wounds were monitored for 14 d. Each bioink scaffold was printed in a cylindrical shape (diameter 6 mm, height 0.6 mm) and applied to the wound. After 3 d of applying 3D scaffolds to wounds, PC- and PCC-bioink-treated mice showed significant reductions in wound size, suggesting that antioxidants in these bioinks promoted tissue regeneration by inducing the conversion of M1 macrophages, which are abundant in chronic wound areas, to M2 macrophages [42]. After 7 d of treatment, wound sizes in the control, TP bioink, and PC bioink groups were 40.0% ± 2.6%, 36.8% ± 2.6%, and 35.3% ± 8.2%, respectively, while the mean wound size in the PCC bioink group was reduced to 23.7% ± 7.9%, suggesting that fibroblasts delivered using PCC bioink promoted wound healing by secreting additional ECM and collagen [43]. Finally, after 14 d of treatment, the mean wound sizes in the PCC, PC, TP, and nontreated control groups were 4.1% ± 1.6%, 5.8% ± 1.4%, 10.8% ± 3.6%, and 16.1% ± 1.8%, respectively (Fig. 8B and C). At this time, no marked difference in mean wound size was observed between the PC and PCC bioink groups. However, on day 7, the PCC bioink group had a significantly smaller wound size than the other groups. These results highlight the potential of 3D scaffolds to effectively codeliver antioxidants and fibroblasts to the wound site, accelerating chronic wound healing in a diabetic mouse model through the synergistic effects of the delivered biologically active molecules. Moreover, the fabricated 3D scaffold provides a moist environment and exhibits potential as an advanced 3D scaffold dressing that can be applied to irregular chronic wound sites with the stable delivery of biologically active molecules. Nevertheless, several challenges remain for future clinical translation, including cost-effective manufacturing, regulatory approval, and the feasibility of applying the bioink in a form suitable for intraoperative or clinical use [44,45]. Once these limitations are overcome, the present PCC-bioink-based 3D scaffold is expected to have promising clinical potential for diabetic chronic wound treatment.

### Histological analysis of tissue regeneration after bioink treatment

**Fig. 9.**
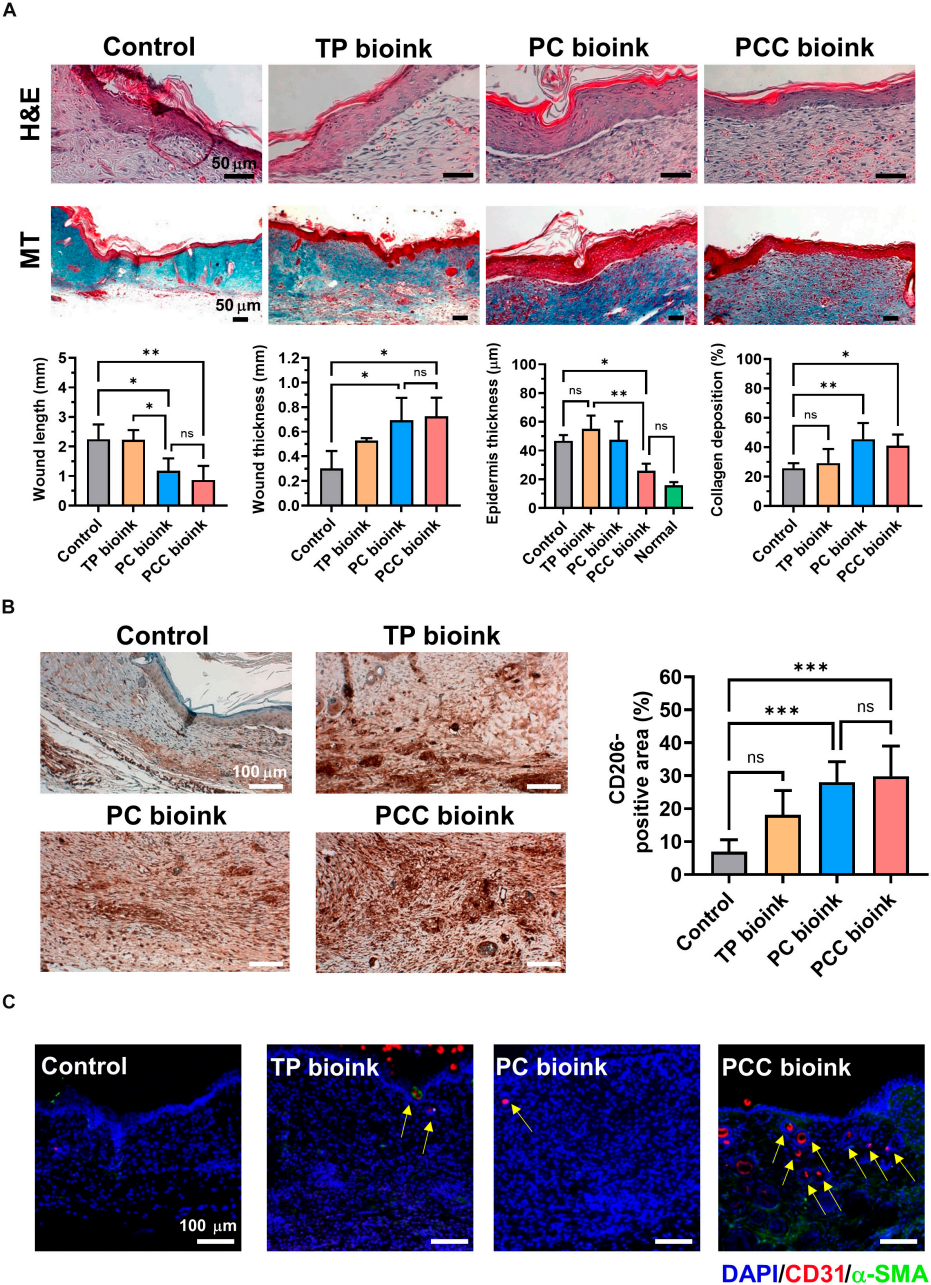
Histological analysis of skin tissues after 14 d of treatment with TP, PC, and PCC bioinks. (A) Hematoxylin and eosin (H&E) staining (×40; scale bar: 50 μm) and Masson’s trichrome (MT) staining (×20; scale bar: 50 μm) and wound lengths, thicknesses, epidermal thicknesses, and collagen deposition (*n* = 4 each group). (B) Immunohistochemical (IHC) staining of CD206 in wound tissues on treatment day 14 (*n* = 4 each group; scale bar: 100 μm). (C) Immunofluorescence (IF) images of CD31 and alpha-smooth muscle actin (α-SMA) in wound tissues on treatment day 14 (scale bar: 100 μm). Quantitation was performed using the ImageJ software, and results are expressed as mean ± standard deviation. DAPI, 4′,6-diamidino-2-phenylindole.

Histological analysis was performed to evaluate the degree of wound healing achieved using the TP, PC, and PCC bioinks. After 14 d of bioink treatment, tissues were collected and analyzed by hematoxylin and eosin staining and MT staining. Marked differences were observed in each group (Fig. 9A). The wound lengths in the nontreated control, TP bioink, and PC bioink groups were 2.24, 2.23, and 1.17 mm, respectively. The PCC bioink treatment group had the smallest wound length of 0.83 mm. The wound thicknesses in the control, TP bioink, PC bioink, and PCC bioink groups were 0.3, 0.53, 0.61, and 0.66 mm, respectively, and the increase in thickness was greatest in the PCC bioink group (Fig. S5), although wound thicknesses in the PC and PCC bioink groups were similar. We propose the following explanation for these: M1 macrophages are abundant in chronic wounds and suppress granulation tissue formation [39], but CA reduced ROS levels and induced differentiation into M2 macrophages, thereby promoting fibroblast proliferation and ECM expression to form thick granulation tissue [46]. Interestingly, hair follicles were observed exclusively in the PCC bioink group, which suggests fibroblast delivery substantially enhanced tissue regeneration compared to antioxidant delivery alone (Fig. S5). In addition, epidermal thicknesses in the PCC bioink group were similar to that of normal tissue, which confirmed that effective regeneration had been achieved. Furthermore, collagen levels in skin tissues, as measured by MT staining, were higher in the PC- and PCC-bioink-treated groups than in the other groups, suggesting that ECM formation was well achieved and that ECM formation was effectively induced by polarization into M2 macrophages and the influence of the delivered fibroblasts [47].

Immunohistochemical staining was performed to evaluate the degree of macrophage polarization in the tissue. Staining was performed using CD206, a representative M2 macrophage marker (Fig. 9B). The brown-stained area (CD206-positive area) was substantially higher in the PCC bioink treatment group than in the control and TP bioink groups. Those of the control and TP bioink groups were 6.9% ± 3.2% and 18.1% ± 6.6%, respectively. On the other hand, the stained areas in the PC and PCC bioink groups were 28.1% ± 2.7% and 38.4% ± 5.3%, respectively, which were significantly higher in the control group. This supports the concept that CA loaded in PC and PCC bioinks has a positive effect on diabetic wound healing by reducing ROS levels in the wound site and promoting macrophage polarization.

In addition to granulation tissue formation and collagen accumulation, the PCC bioink promoted angiogenesis. Immunofluorescence staining with α-SMA and CD31 showed no vascular staining in the control group, whereas a small number of blood vessels were stained in the TP and PC bioink groups. In contrast, a large amount of vascular staining was observed in the PCC bioink group (Fig. 9C). This result is thought to be due to the formation of α-SMA-positive mature blood vessels due to the increase in the number of M2 macrophages inside the damaged tissue [39]. In addition, the delivered fibroblasts may have supported angiogenesis through ECM proliferation and promoted the proliferation and migration of vascular endothelial cell growth factors such as vascular endothelial growth factor and fibroblast growth factor [48]. These results demonstrate that PCC bioink can effectively contribute to chronic wound healing through macrophage polarization and fibroblast delivery.

Through in vitro and in vivo experiments, 3D printed scaffolds utilizing PCC bioinks demonstrated notable biological activities, including antioxidant effects, M2 macrophage polarization, and angiogenesis. Notably, the proposed bioink exhibits high-resolution printability and structural stability, which are advantageous for producing customized scaffolds and offer practical benefits for application to actual wound sites. Among previously studied 3D scaffolds for diabetic wound treatment, bioinks utilizing MoS_2_ nanosheets and dextran have been shown to exhibit strong ROS scavenging ability and antibacterial effects but face limitations such as low printing resolution due to poor mechanical properties and the requirement of external near-infrared stimulation for effective wound healing [49]. Another example is antibiotic-loaded polycaprolactone-based scaffolds, which provide excellent mechanical properties and high printing resolution; however, they suffer from an initial burst release of antibiotics and lack biological functions related to tissue regeneration [50]. In contrast, the PCC bioink system developed in this study maintains the structure of high-resolution 3D scaffolds even under physiological conditions through physical cross-linking. In addition, the incorporation of CA and fibroblasts enables simultaneous therapeutic functions. These results demonstrate the potential of the PCC bioink as a multifunctional scaffold with both mechanical stability and therapeutic efficacy.

## Conclusion

We propose a thermo-responsive bioink with an antioxidant effect that can directly deliver cells to wounds. Our goal was to use a 3D scaffold customized to an irregular wound shape to promote rapid wound closure and effective tissue regeneration and accelerate the healing of chronic wounds, especially diabetic wounds. CA encapsulated in the hydrogel was stably loaded due to π–π interaction and hydrophobic interaction, and the resulting PC bioink formed a high-density hydrogel network with excellent mechanical strength. In addition, the thermo-responsive property of the bioink allowed for the control of mechanical properties by adjusting the printer nozzle temperature without an additional cross-linking process, resulting in excellent extrudability and printability. This enabled high-resolution scaffold printing, and the printed scaffold maintained its shape for 30 d in a physiological environment due to its excellent mechanical properties at body temperature. In particular, the CA that loaded in the PC bioink was stably encapsulated inside the bioink through various physical interactions and was sustained and long-term released to effectively remove ROS in chronic wound tissues and promote the M2 polarization of macrophages and tissue regeneration. In addition, we prepared PCC bioink loaded with fibroblasts in PC bioink for more effective diabetic wound healing, and the scaffold made with PCC bioink still maintained a high-resolution scaffold shape with high cell viability for 7 d. When PCC bioink was applied to a diabetic mouse wound model, wound closure was substantially accelerated and high tissue regeneration ability was observed. In addition, due to its excellent biocompatibility and biodegradability, it was possible to release various bioactive molecules without any side effects in in vivo application. This paper suggests the potential of PCC bioink as a next-generation bioink platform for diabetic wound healing. The proposed thermo-responsive bioink technology is expected to be utilized for various tissue regeneration treatments, not only for diabetic wounds but also for rheumatoid arthritis and ischemic diseases. Furthermore, overcoming limitations such as scaled-up production of bioink and regulatory approval will further increase the potential for clinical translation.

## Data Availability

All data obtained during the study are included in the article or uploaded as Supplementary Materials. Data that support the findings of this research are available from the corresponding authors upon receipt of reasonable request.
