# Supplementary material for: Thermo-responsive Bioink for Personalized 3D Printed Scaffolds with Antioxidant and Fibroblast Delivery to Accelerate Diabetic Wound Healing
Source: Biomater Res. 2025 Jun 11;29:0216. doi: 10.34133/bmr.0216 (PMC12352585; doi:10.34133/bmr.0216)
Supplement: Supplementary 1 — Supplementary Materials Figs. S1 to S5 Table S1 [file bmr.0216.f1.zip › Revised_Supplementary Materials.docx]

Supplementary Materials

**Thermo-Responsive Bioink for Personalized 3D-Printed Scaffolds with Antioxidant and Fibroblast Delivery to Accelerate Diabetic Wound Healing**

Jisun Kim^1, 2‡^, Jiyeon Lee^4‡^, Jung-Kyo Cho^3^, Ki Wan Bong^2^, Soo-Chang Song^1, 3 ,4*^

^1^ Center for Biomaterials, Biomedical Research Institute, Korea Institute of Science and Technology, Seoul, 02792, Republic of Korea

^2^ Department of Chemical and Biological Engineering, Korea University, Seoul 02841, Republic of Korea

^3^ Nexgel Biotech, Co., Ltd., Hanam 12939, Republic of Korea

^4^ Division of Bio-Medical Science & Technology, KIST school, University of Science and Technology, Seoul 02792, Republic of Korea

*Corresponding author

Soo-Chang Song, Ph.D.

Center for Biomaterials, Biomedical Research Institute, Korea Institute of Science and Technology, Seoul, 02792, Republic of Korea

Nexgel Biotech, Co., Ltd., Hanam 12939, Republic of Korea

E-mail: scsong@kist.re.kr

Tel: +82-2-958-5123

**Additional experimental details**

**Synthesis to α-amino-ω-methoxy poly(ethylene glycol) (AMPEG).**

750 Da Methoxy poly(ethylene glycol) was dried at 45 ℃ under vacuum condition for 1day. The first step is the synthesis of Tosyl-PEG-CH_3_. Briefly, Methoxy poly(ethylene glycol) (MW 750 Da, 406 g, 0.54 mol), tosyl chloride (102 g, 0.54 mol), and TEA (109.5 ml, 1.08 mol) were dissolved in chloroform (1000 mL) and stirred in an ice bath for 24 h. The solution was then purified by filtration and evaporation. The obtained product was separated with chloroform and distilled water. The chloroform layer was collected, magnesium sulfate was added to the purified product solution and stirred for 3 hours to remove moisture, and tosyl-PEG-CH_3_ was obtained by filtration and evaporation. The obtained tosyl-PEG-CH_3_ (412 g, 0.45 mol) was dissolved in ACN (1000 mL) containing sodium azide (59 g, 0.91 mol) and reacted at 90-100°C for 48 hours under N_2_ atmosphere. N_3_-PEG-CH_3_ was obtained using the same purification steps as described above. Finally, obtained N_3_-PEG-CH_3_ (307 g, 0.40 mol) was dissolved in THF (1000 mL) containing triphenylphosphine (114 g, 0.44 mol) and distilled water (10.7 ml, 0.59 mol) and stirred at RT for 24 h. The solution was then evaporated and precipitated in 1000 mL of distilled water in a cold chamber for 24 h. The precipitate was filtered, separated with ethyl ether three times. The distilled water layer containing the product was collected and evaporated to remove residual solvents. Finally, the obtained AMPEG was stored at 0-4 °C.

**Synthesis of PPZ.**

IleOEt and AMPEG were dried under vacuum at 40 °C for 3 days before synthesis started. THF and TEA were prepared under anhydrous conditions in an N_2_ atmosphere. hexachlorocyclotriphosphazene (10 g) and AlCl_3_ (0.5 g) were mixed in a glass ampule under an Ar atmosphere inside a glove box. To synthesize poly(dichlorophosphazene), the ampule was sealed and heated at 250 °C for 5 hours. The synthesized poly(dichlorophosphazene) weighed and was sealed with a septum. The entire synthesis was performed under N_2_ using a cannula and Schlenk line to prevent moisture ingress. Dry IleOEt·HCl (24.82 g, 0.126 mol) was dissolved in 200 ml of anhydrous THF and 30 minutes later, 100 ml of TEA was added. The reactor was then placed in a dry ice bath. The reaction was initiated by adding poly(dichlorophosphazene) (10 g) dissolved in THF to the reactor using a cannula. The reaction was allowed to proceed at room temperature for 3 hours, and then the temperature was increased to 45 °C for 1 day. After the reaction, Aminoethanol (1.31 ml, 0.021 mol) was added to the reaction flask. After 30 minutes, AMPEG (27.18 g, 0.036 mol) dissolved in 50 ml of anhydrous THF was added using a canular. The reaction flask was reacted at 50 °C for 1 day. The product was purified by a glass filter and evaporator to remove solvent and side products, and then using an n-hexane precipitation process for 1 day. After purification, the obtained product was dialyzed in methanol for 4 days, followed by dialysis in deionized water for another 4 days using a dialysis bag (MWCO: 12–14 kDa). The obtained polymer solution was filtered using a syringe filter (PP, 0.45 μm pore size) and freeze-dried to remove water.

**Table S1.** RT-PCR primer sequence

| **Gene** | **Primer (forward)** | **Primer (reverse)** |
| --- | --- | --- |
| β-actin | TCCACCTTCCAGCAGATG | CTCAGTAACAGTCCGCCTA |
| TNF-α | ACGCTCTTCTGTCTACTGA | CTTGGTGGTTTGCTACGA |
| iNOS | GTCCTACACCACACCAAA | CTCCAATCTCTGCCTATCC |
| Arg-1 | CTGAAGGAACTGAAAGGAAAG | GTCTCGCAAGCCAATGTA |
| IL-10 | CTGCTAACCGACTCCTTA | AATGCTCCTTGATTTCTGG |


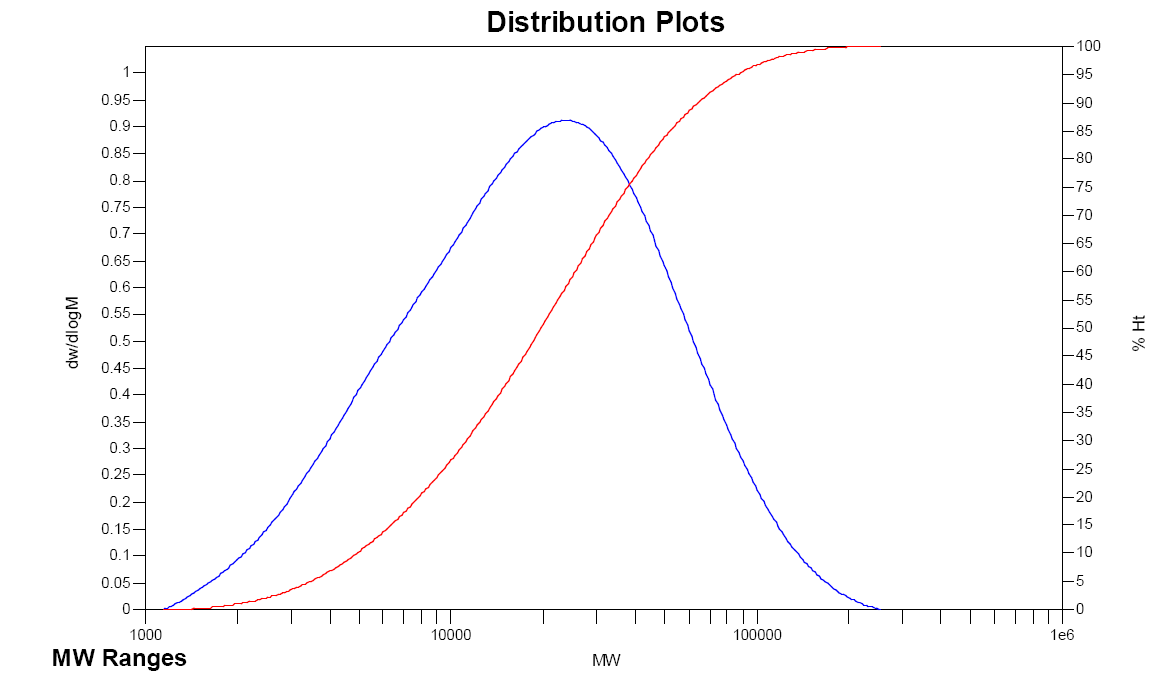


| Mp (g/mol) | Mn (g/mol) | Mw (g/mol) | Mz (g/mol) | PDI |
| --- | --- | --- | --- | --- |
| 23782 | 11493 | 28561 | 56530 | 2.4851 |

**Figure S1.** GPC analysis of synthesized Tyr-PPZ polymer. Mp: peak molecular weight; Mn: number average molecular weight; Mw: weight average molecular weight; Mz: Z average molecular weight; PDI: polydispersity index.


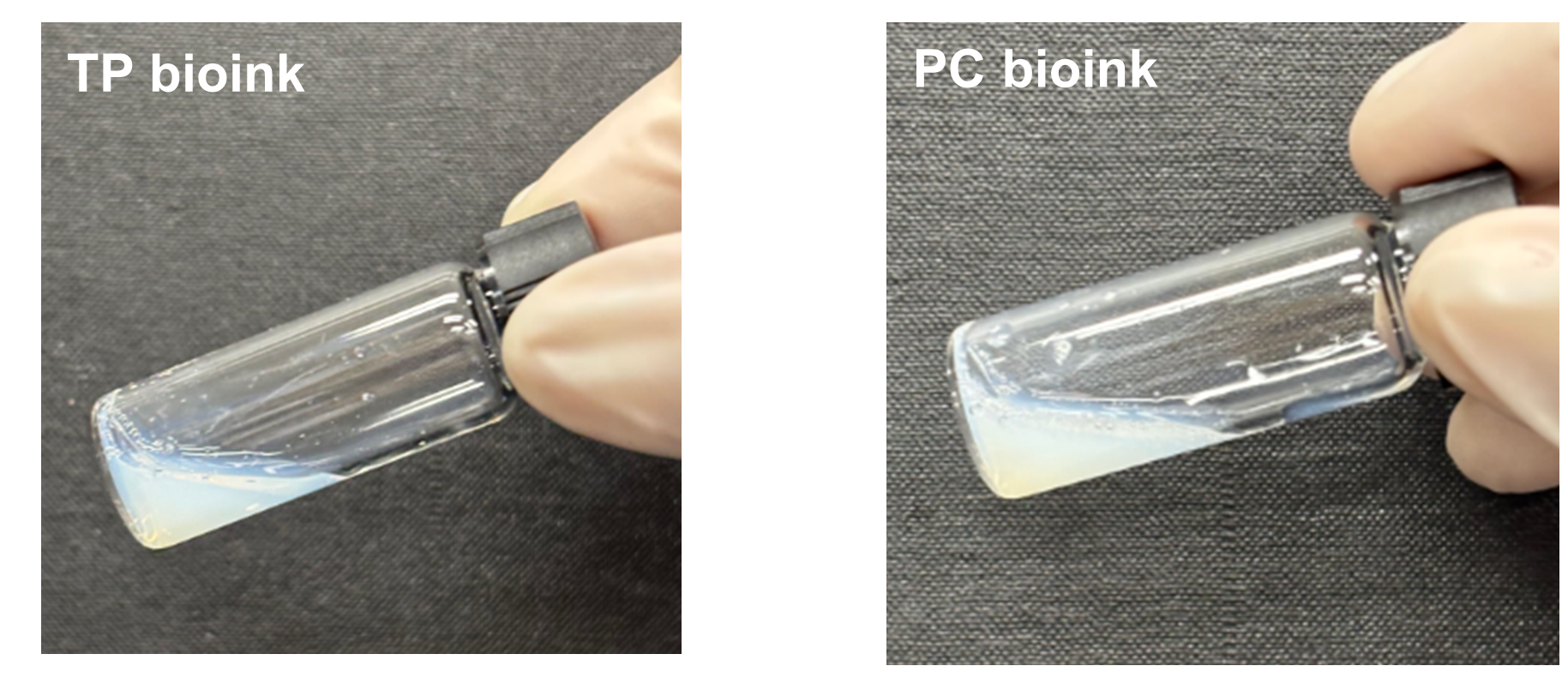


**Figure S2.** Representative image of TP bioink and PC bioink.


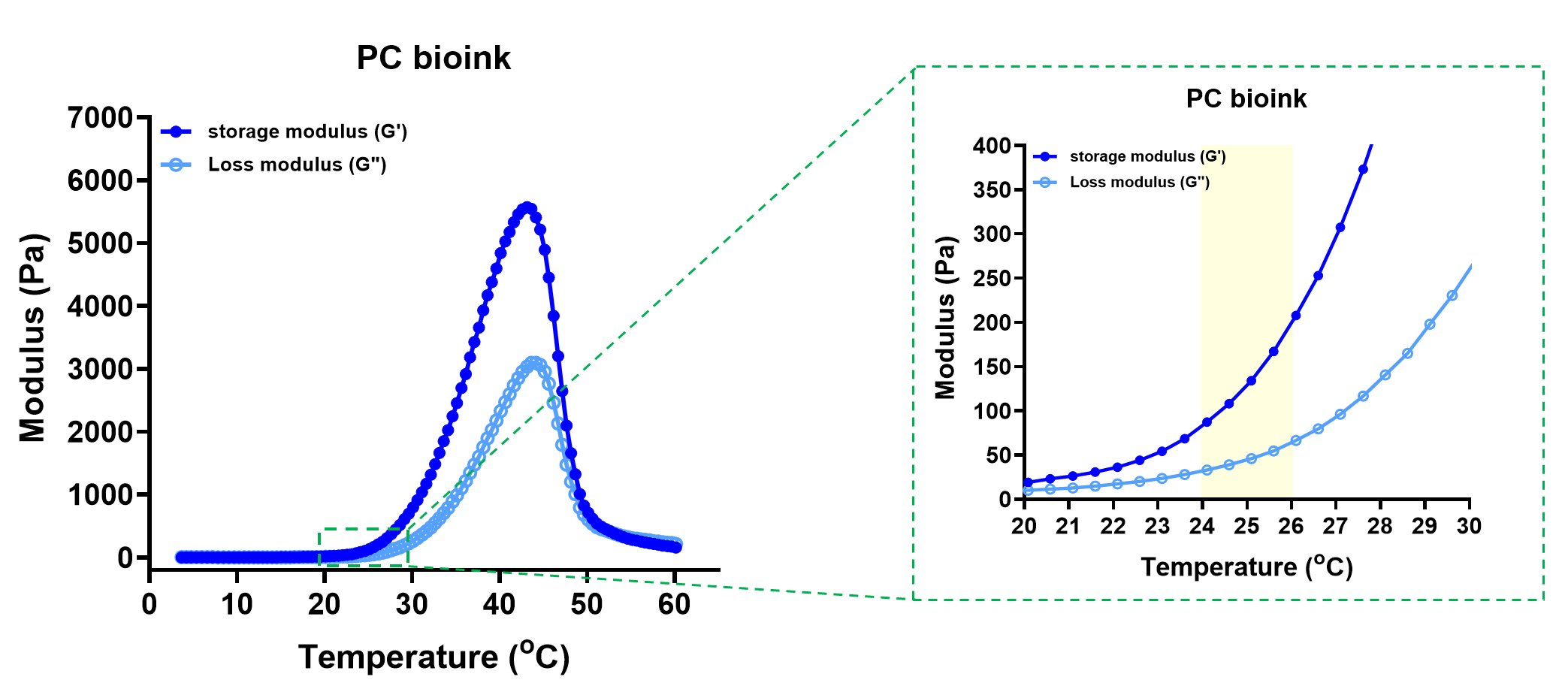


**Figure S3.** Detailed rheological behavior of PC Bioink between 20-30°C within the full temperature range


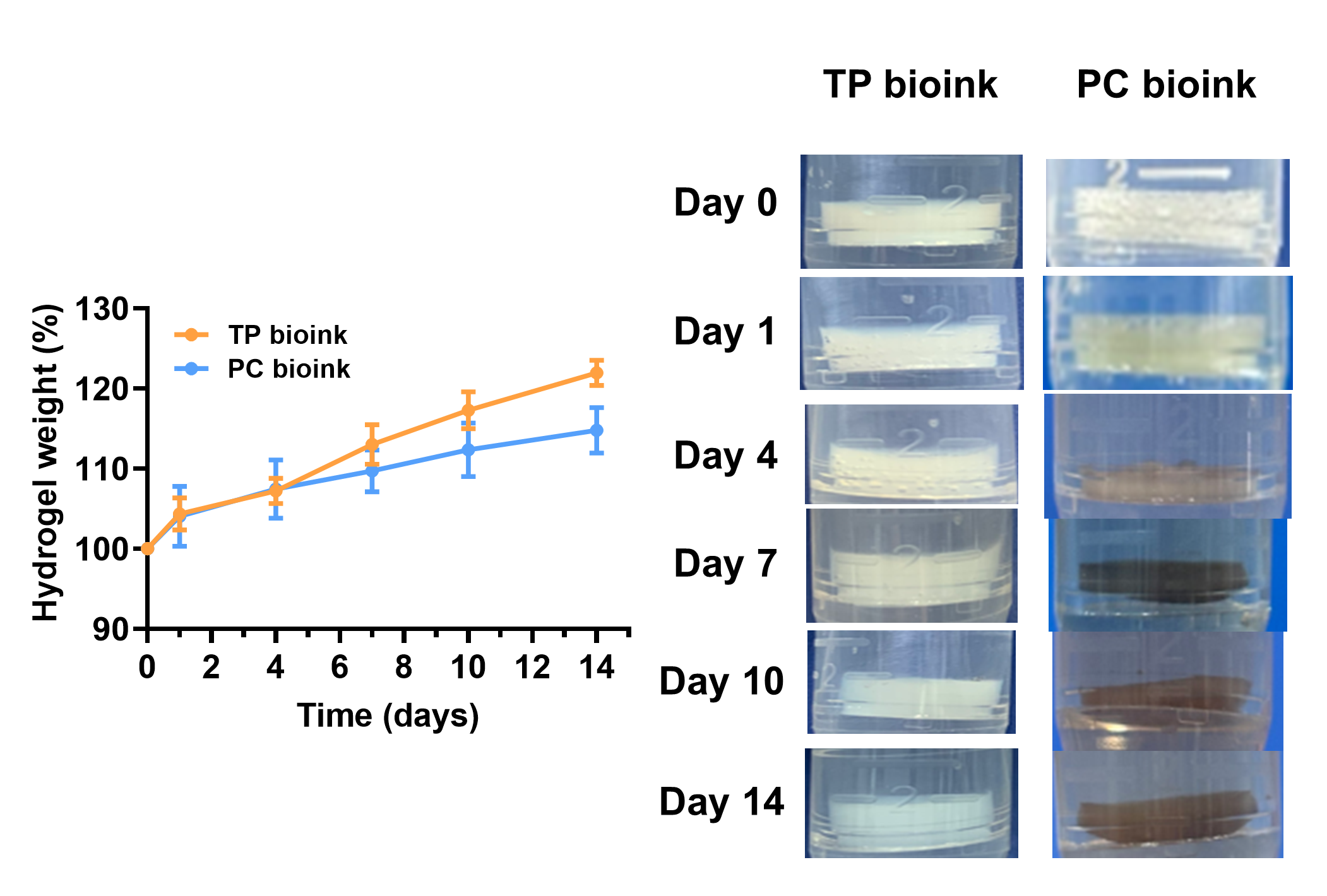


**Figure S4.** Hydrogel weight change of TP bioink and PC bioink (100 μL loaded per Millicell insert) after 14 days of incubation in PBS at 37 °C.


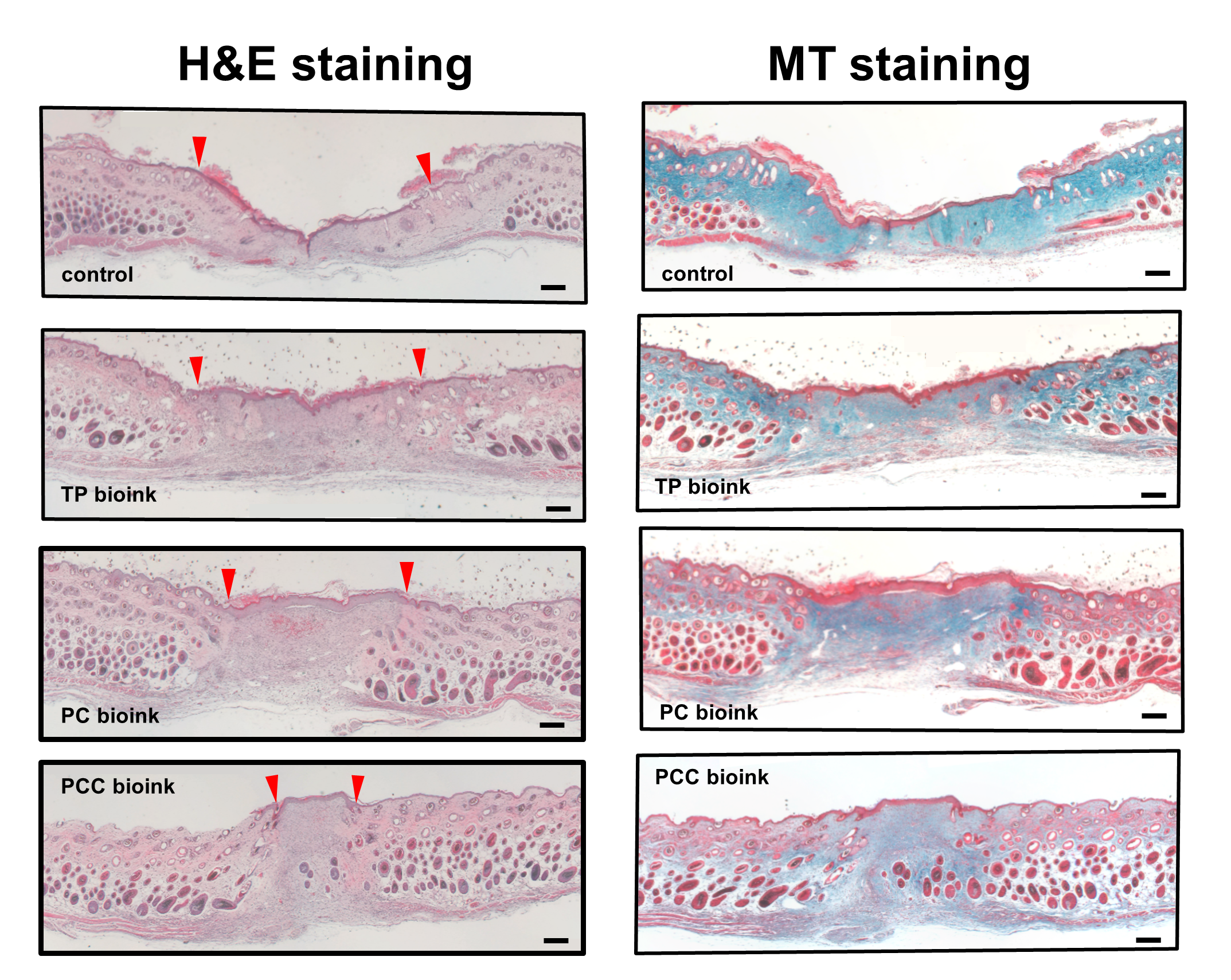


**Figure S5.** H&E and MT staining images of overall diabetic wound tissues harvested 14 days after bioink treatment (scale bar: 200 μm).
